# Measure transcript integrity using RNA-seq data

**DOI:** 10.1186/s12859-016-0922-z

**Published:** 2016-02-03

**Authors:** Liguo Wang, Jinfu Nie, Hugues Sicotte, Ying Li, Jeanette E. Eckel-Passow, Surendra Dasari, Peter T. Vedell, Poulami Barman, Liewei Wang, Richard Weinshiboum, Jin Jen, Haojie Huang, Manish Kohli, Jean-Pierre A. Kocher

**Affiliations:** Division of Biomedical Statistics and Informatics, Mayo Clinic, Rochester, MN 55905 USA; Department of Oncology, Mayo Clinic, Rochester, MN 55905 USA; Department of Molecular Pharmacology and Experimental Therapeutics, Mayo Clinic, Rochester, MN 55905 USA; Department of laboratory medicine and pathology, Mayo Clinic, Rochester, MN 55905 USA; Department of Biochemistry and Molecular Biology, Mayo Clinic, Rochester, MN 55905 USA

**Keywords:** Transcript integrity number, TIN, RNA-seq quality control, Gene expression

## Abstract

**Background:**

Stored biological samples with pathology information and medical records are invaluable resources for translational medical research. However, RNAs extracted from the archived clinical tissues are often substantially degraded. RNA degradation distorts the RNA-seq read coverage in a gene-specific manner, and has profound influences on whole-genome gene expression profiling.

**Result:**

We developed the transcript integrity number (TIN) to measure RNA degradation. When applied to 3 independent RNA-seq datasets, we demonstrated TIN is a reliable and sensitive measure of the RNA degradation at both transcript and sample level. Through comparing 10 prostate cancer clinical samples with lower RNA integrity to 10 samples with higher RNA quality, we demonstrated that calibrating gene expression counts with TIN scores could effectively neutralize RNA degradation effects by reducing false positives and recovering biologically meaningful pathways. When further evaluating the performance of TIN correction using spike-in transcripts in RNA-seq data generated from the Sequencing Quality Control consortium, we found TIN adjustment had better control of false positives and false negatives (sensitivity = 0.89, specificity = 0.91, accuracy = 0.90), as compared to gene expression analysis results without TIN correction (sensitivity = 0.98, specificity = 0.50, accuracy = 0.86).

**Conclusion:**

TIN is a reliable measurement of RNA integrity and a valuable approach used to neutralize in vitro RNA degradation effect and improve differential gene expression analysis.

**Electronic supplementary material:**

The online version of this article (doi:10.1186/s12859-016-0922-z) contains supplementary material, which is available to authorized users.

## Background

In vitro RNA degradation occurs in most of the isolated RNA samples and the degree of degradation depends on the specimen collection and storage conditions such as formalin-fixed, paraffin-embedded (FFPE) and fresh frozen [1–3]. This is especially a major issue for clinical tissues collected in surgery suites because optimal storage of collected specimens is often not the primary focus in that setting. There have been multiple studies showing that in vitro degradation of RNA impairs accurate measurement of in vivo gene expression [4, 5]. RNA degradation has not been a major problem up to recently since it has a minor influence on gene expression measured with hybridization-based microarray platforms, in which the expression of each gene is measured by only a few short, discrete probes. For example, a previous study found that only 0.67 % (275 out of 41,000) of the probes were significantly affected by in vitro RNA degradation [6]. However, in recent years, more studies including The Cancer Genome Atlas consortium (TCGA) are switching to use sequencing-based RNA-seq to profile gene expression. RNA-seq works under the assumption that every nucleotide of the transcript has the equal chance to be sequenced and the amount of reads produced from a transcript is proportional to the abundance and length of the transcript. However, if RNA molecules were partially or completely degraded the corresponding read yield would be also distorted accordingly. Hence, in vitro RNA degradation introduces a major source of variation when measuring gene expression via RNA-seq. In support of this hypothesis, a recent study found that up to 56 % of the genes were differentially expressed due to in vitro RNA degradation [5].

RNA Integrity Number (RIN) is the most widely used approach to assess in vitro RNA degradation [1–3, 7]. However, the RIN metric has several weaknesses that limit its applications in both pre-sequencing RNA sample screening and post-sequencing RNA-seq data analysis. First, the RIN score relies heavily on the amount of 18S and 28S ribosome RNAs; the four main features used by the RIN algorithm includes the “total RNA ratio”, “28S-region height”, “28S area ratio” and the “18S:28S ratio”. While this metric accurately captures the integrity of ribosomal RNAs, it fails to measure the mRNA integrity directly, which is the main input for RNA sequencing. Second, RNA decay rate is transcript specific and it is modulated by several endogenous and exogenous factors as well as other factors including “AU-rich” sequence, transcript length, GC content, secondary structure, RNA-protein complex [4, 5]. It was found that RNA decay rate varies between functional groups [6, 8] and between transcripts by up to ten-fold [5, 9, 10]. Third, RIN is an overall assessment of RNA quality and cannot be used as a co-factor to adjust for differential RNA degradation between transcripts in downstream gene expression analysis. Finally, it has been reported that RIN was not a sensitive measure of RNA quality for substantially degraded samples (https://www.illumina.com/content/dam/illumina-marketing/documents/products/technotes/evaluating-rnaquality-from-ffpe-samples-technical-note-470-2014-001.pdf). Illumina® proposed DV_200_ metric (the percentage of RNA fragments > 200 nucleotides) to assess RNA quality. However, similar to RIN, DV_200_ is also an overall measurement and fails to determine RNA degradation at transcript level.

The reduction of sequencing cost has opened doors for large-scale, RNA-seq-based, gene expression profiling studies (like TCGA) that use clinical specimens with rich outcomes data. At the same time, the RNA quality of these clinical samples could vary significantly and poses a great challenge to gene expression analysis. Here we developed a novel algorithm–transcript integrity number (TIN)–to evaluate RNA integrity from RNA-seq data. We applied our TIN algorithm to RNA-seq data generated from 12 human glioblastoma (GBM) cell line samples, 20 human peripheral blood mononuclear cell samples (PBMC), and 120 metastatic castration resistant prostate cancer (mCRPC) samples. Our results showed that TIN metric accurately measured the mRNA integrity at transcript level, as demonstrated by high concordance with RNA fragment size that estimated from RNA-seq read pairs. We also demonstrated that the median TIN score (medTIN) across all transcripts can be an accurate and reliable measurement of RNA integrity at transcriptome (or “sample”) level. More importantly, the TIN that is computed for each transcript can be used to adjust gene expression and improve differential expression analysis by reducing the false positives ascribed to in vitro RNA degradation.

## Results and discussion

### Measuring sample level RNA integrity

**Fig. 1:**
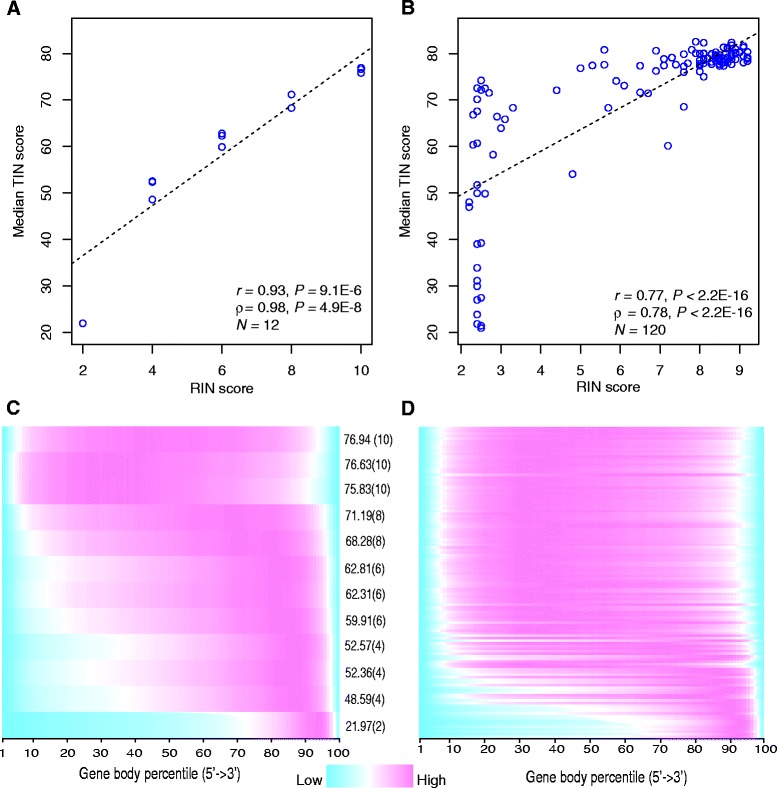
Evaluating median TIN score (medTIN) metric using RIN and gene body read coverage. **a** Scatterplot showing correlation between the medTIN and the corresponding RIN score for 12 GBM samples. Black dashed line is the linear regression line fitted to data. **b** Scatterplot showing correlation between the medTIN and the corresponding RIN score for 120 mCRPC samples. Black dashed line is the linear regression line fitted to data. **c** Gene body coverage profiles for 12 GBM samples. Samples were ranked from top to bottom on the y-axis in the decreasing order of medTIN. Numbers in parentheses are the corresponding RIN scores. **d** Gene body coverage profiles for 120 mCRPC samples. Samples were ranked from top to bottom on the y-axis in the decreasing order of medTIN. *r* stands for Pearson’s correlation coefficient; ρ stands for Spearman’s correlation coefficient

We used the median TIN score (medTIN) of all the transcripts to measure the overall RNA integrity of a sample. We evaluated the concordance between medTIN and the widely used RIN metric using three independent human datasets: GBM cell lines, PBMCs, and mCRPC. Each of these datasets has samples covering a broad range of RIN values. GBM samples have RIN values ranging from 2 to 10 (Additional file 1: Table S1), PBMC samples have RIN values ranging from 2.8 to 9.4 (Additional file 2: Table S2) and mCPRC samples have RIN values ranging from 2.2 to 9.2 (Additional file 3: Table S3). The Pearson correlation coefficients between medTINs and the corresponding RIN scores for the GBM, mCRPC and PBMC samples were 0.93 (*P* = 9.1 × 10^−6^; Fig. 1a), 0.77 (*P* < 2.2 × 10^−16^; Additional file 4: Figure S1) and 0.83 (*P* = 7.3 × 10^−6^; Fig. 1b), respectively. The high concordance highlighted that medTIN was a reliable index of the overall RNA quality of a sample. Compared to GBM samples. The correlation between RIN and medTIN in mCRPC samples was lower, which was probably because the RIN scores were clustered into two extremes: with 28 (23.3 %) samples had RIN < 3 and 61 (50.8 %) samples had RIN > 8 (Fig. 1b, Additional file 5: Figure S2, Additional file 3: Table S3).

The 3′ bias observed in RNA-seq data could arise from RNA degradation by 5′ exonuclease [11, 12], and the commonly used polyA enrichment approach would lead to a even stronger 3′ bias particularly in degraded RNA samples because oligo (dT) selection will only isolate the most 3′ portion of the transcript [13]. Consistently with this hypothesis, we found that samples with lower medTIN score usually had more skewed gene body coverage (Fig. 1c-d). The PBMC dataset was excluded from further analysis because its single-end sequencing design prevents the estimation of RNA fragment size.

**Fig. 2:**
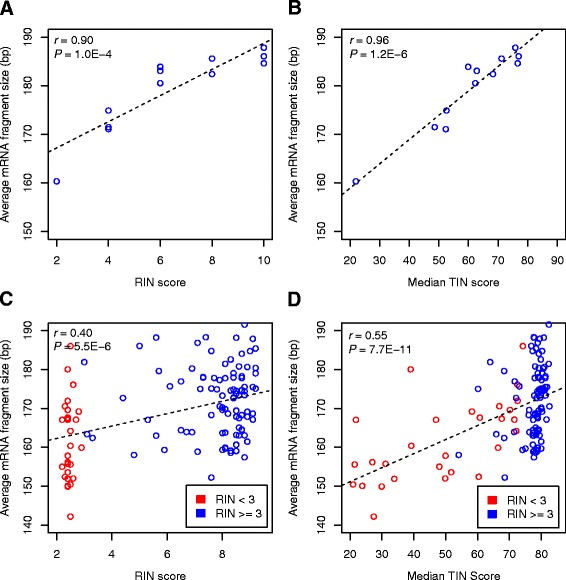
Evaluating median TIN score (medTIN) and RIN metric using sample level average RNA fragment size. The average RNA fragment size of a sample was estimated from all read pairs that uniquely mapped to the reference genome (see Methods). **a** Correlation between RIN score and the average RNA fragment size for 12 GMB samples. **b** Correlation between medTIN and average RNA fragment size for 12 GMB samples. **c** Correlation between RIN score and average RNA fragment size for 120 mCRPC samples. **d** Correlation between medTIN and average RNA fragment size for 120 mCRPC samples. (**c**-**d**) Samples with RIN < 3 and RIN ≥3 were indicated as red and blue circles, respectively. (**a**-**d**) Linear regression lines fitted to data are indicated as black dashed lines

The average RNA fragment size of a sequencing library, which can be directly estimated from mapped read pairs, is a surrogate measurement of RNA integrity because RNA fragments become smaller after in vitro degradation process. We therefore computed the average RNA fragment size of all read pairs to measure the integrity of a RNA sample, and compared it with medTIN and RIN metrics, respectively. For the 12 GBM samples, both RIN (*r* = 0.90, *P* = 1.0 × 10^−4^; Fig. 2a) and medTIN (*r* = 0.96, *P* = 1.2 × 10^−6^; Fig. 2b) were strongly correlated with the average RNA fragment sizes with medTIN metric performed slightly better. For mCRPC RNA samples, the medTIN (*r* = 0.55, *P* = 7.7 × 10^−11^) also performed significantly better than RIN (*r* = 0.40, *P* = 5.5 × 10^−6^) (Fig. 2c, d). We further evaluated the performance of medTIN metric on severely degraded samples using a subset of 28 mCRPC samples that have RIN values < 3 (Additional file 3: Table S3). We observed no positive correlation between RIN and the corresponding average RNA fragment sizes (*r* = 0.089, *P* = 0.65; Additional file 6: Figure S3a). In contrast, we observed a strong positive correlation between medTINs and the RNA fragment sizes for these samples (*r* = 0.62, *P* = 4.5 × 10^−4^; Additional file 6 Figure S3b). These results highlighted medTIN was more sensitive than RIN to measure the integrity of RNA samples that were severely degraded.

### Measuring transcript level RNA integrity

**Fig. 3:**
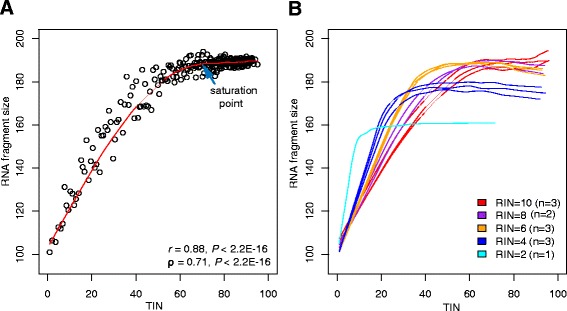
Evaluating TIN metric using transcript level RNA fragment size. The average RNA fragment size (y-axis) of a particular transcript was estimated from all the read pairs that uniquely mapped to the transcript (see Methods). **a** Correlation between TIN score and transcript level RNA fragment size. A single GBM sample (SRR873822; RIN = 10) was used to produce the figure. Each dot represents 50 transcripts. Red curve indicates the locally weighted polynomial regression curve. **b** Locally weighted polynomial regression curves for all GBM RNA-seq samples

Compared to RIN and other global measurements [14–16], one of the major improvements of TIN is to measure RNA integrity of individual transcripts/genes. We evaluated the performance of TIN by correlating it with the transcript level average RNA fragment size. As shown in Fig. 3a, TIN score and RNA fragment size had a strong positive correlation (Pearson’s *r* = 0.88, *P* < 2.2 × 10^−16^; Spearman’s *ρ* = 0.71, *P* < 2.2 × 10^−16^) suggesting that TIN was a good metric to measure transcript integrity. Interestingly, we found the average RNA fragment size became asymptotically stable as TIN score went beyond certain threshold (i.e. saturation point). For instance, in Fig. 3a, the saturation point was around TIN = 70, and the correlation between TIN and RNA fragment size was much higher for transcripts with TIN < 70 (*r* = 0.94, *P* < 2.2 × 10^−16^) than that of transcripts with TIN > 70 (*r* = 0.22, *P* = 0.003). We observed the similar trend in all GBM samples with different RIN values (Fig. 3b, Additional file 7: Figure S4). This is because the RNA degradation is not the sole determinant for RNA fragment size as most sequencing library preparation protocols also incorporate a RNA (or cDNA) “fragmentation step”. Therefore, the sizes of RNA fragments of a particular transcript are determined by two factors at the same time: the fragmentation intensity during library preparation and the RNA degradation. Presumably, transcripts with larger TIN values had better RNA integrity and therefore “fragmentation step” played a dominant role in determining the fragment size whereas RNA degradation played a major role in affecting the fragment size of transcripts with lower TIN values.

As the overall RNA quality decreased, concordance between TIN and fragment size was also decreased (Additional file 8: Figure S5). For example, the Pearson’s *r* were 0.88, 0.89, and 0.88 for three samples with RIN score of 10 whereas the Pearson’s *r* were 0.66, 0.61 and 0.63 for three samples with RIN score of 6 (Additional file 7: Figure S4 and Additional file 8: Figure S5). This is because the non-linear relationship between TIN and the RNA fragment size (Fig. 3), and the correlation was mainly determined by those transcripts whose TINs were smaller than saturation point.

### Effects of transcript features on TIN score

**Fig. 4:**
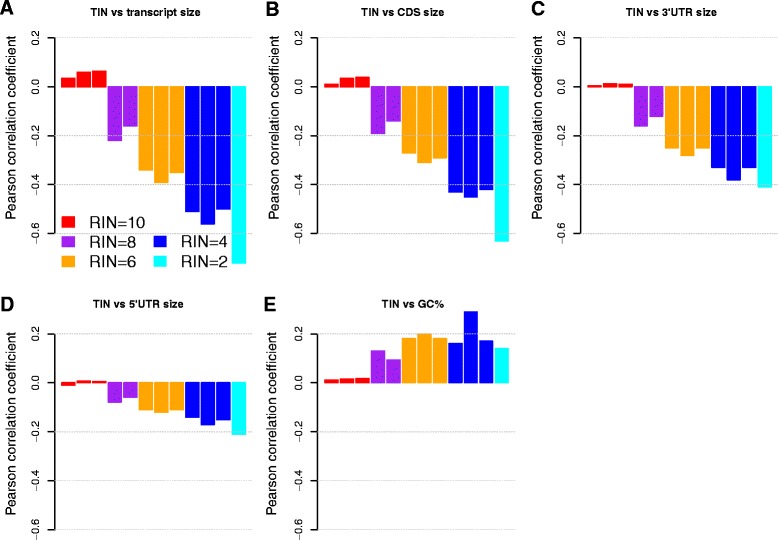
Correlation between TIN score and transcript features including (**a**) transcript size, (**b**) CDS size, (**c**) 3′UTR size, (**d**) 5′UTR size and (**e**) GC content. The GBM dataset was used to make these comparisons. CDS stands for coding DNA sequence. UTR stands for un-translated region

We demonstrated that medTIN and TIN were useful metrics for assessing the RNA integrity at sample and individual transcript level, respectively. Next, we asked what characteristics of transcripts could affect the RNA degradation and thereby affect TIN score. To accomplish this, we compared the mRNA size, CDS (Coding DNA Sequence) size, 5′UTR (5-prime Untranslated Region) size, 3′UTR size and GC content of the transcripts to their corresponding TIN scores. We found no or very weak correlation between transcript size and TIN score in samples with high RNA integrity. However, we observed a strong negative correlation between the transcript size and TIN score for samples with lower RNA quality (Fig. 4a; Additional file 9: Figure S6). For example, the Pearson’s *r* was 0.035, 0.059 and 0.063 for three GBM samples with RIN of 10 whereas the Pearson’s *r* was -0.50, -0.51 and -0.56 for three GBM samples with RIN of 4. The Pearson’s *r* was -0.72 for a sample with RIN value of 2. We observed similar trends for CDS size (Fig. 4b; Additional file 10: Figure S7), 3′UTR size (Fig. 4c; Additional file 11: Figure S8) and 5′UTR size (Fig. 4d; Additional file 12: Figure S9). However, these features had weaker association with TIN when compared with that of the transcript size. The observation that larger transcripts had lower TIN scores in degraded samples suggested these transcripts were more susceptible to the in vitro degradation process. In contrast to transcript size that had negative correlation with TIN score, the GC content had positive albeit weak correlation with TIN score, suggesting GC-rich transcripts were resistant to RNA degradation (Fig. 4e; Additional file 13: Figure S10). This could be explained by the fact that GC base pairings are more stable than AU base pairings and transcripts with high GC content tend to have better thermodynamic stability. A similar observation was also made by another study [17].

### Using TIN to adjust for RNA degradation in gene differential expression analysis

**Fig. 5:**
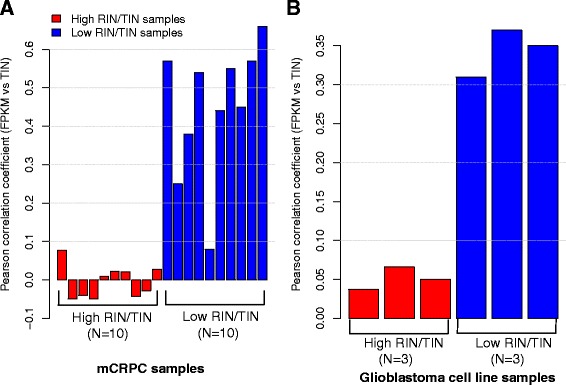
Comparing Pearson correlation coefficient between gene expression (FPKM) and TIN score. **a** 20 mCRPC samples with 10 high RIN/TIN samples (RIN_mean_ = 7.1, RIN_sd_ = 1.6; red bars) and 10 low RIN/TIN samples (RIN_mean_ = 2.4, RIN_sd_ = 0.08; blue bars). **b** 6 GBM samples with 3 high RIN/TIN samples (red bars) and 3 low RIN/TIN samples (blue bars). FPKM stands for Fragments Per Kilobase of transcript per Million mapped reads

We first investigated if TIN metric was useful to improve gene differential expression analysis and reduce false positives. We selected 10 mCRPC samples with lower RIN (RIN_mean_ = 2.4, RIN_sd_ = 0.08) values and another 10 samples with higher RIN (RIN_mean_ = 7.1, RIN_sd_ = 1.6) values (Additional file 14: Table S4). All of these samples were biopsied from bone metastases and processed using the same protocol. As an independent dataset, we also selected 3 GBM samples with RIN value of 10 and 3 samples with RIN value of 4. We found that the normalized gene expression count (FPKM) did not correlate with the corresponding TIN scores in mCRPC samples with relatively higher RNA quality (Fig. 5a; Additional file 15: Figure S11A-J). However, FPKM values positively correlated with TIN scores in mCRPC samples with lower RNA quality (Fig. 5a; Additional file 15: Figure S11K-T). We could reproduce this result using the GBM data (Fig. 5b; Additional file 16: Figure S12). It is notable that the expression fold change between the high RIN and the low RIN samples was also significantly correlated with the TIN fold change; the Pearson’s *r* were 0.45 (*P* < 2.2 × 10^−16^) and 0.64 (*P* < 2.2 × 10^−16^) for mCRPC and GBM data, respectively (Additional file 17: Figure S13).

**Fig. 6:**
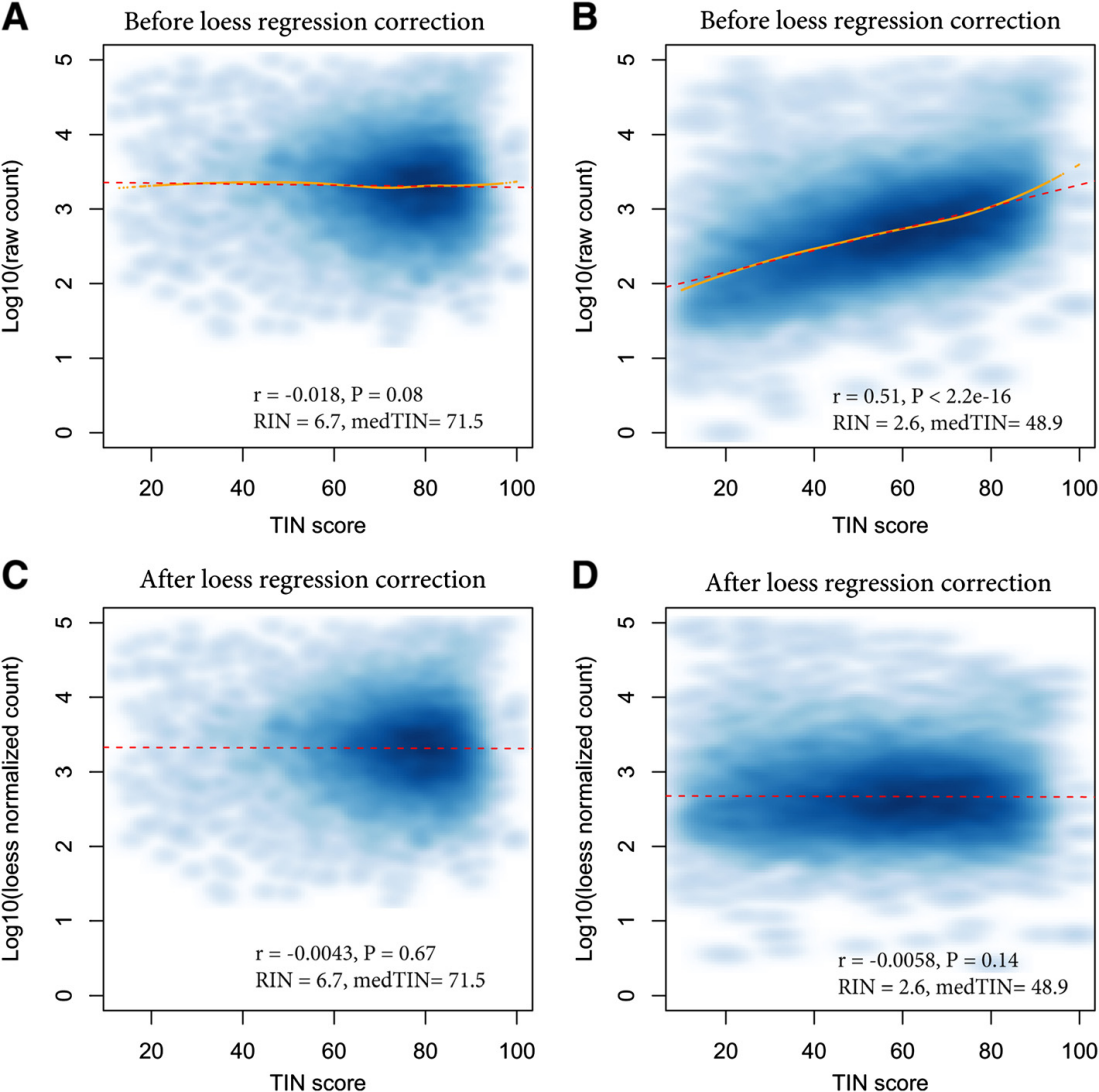
Evaluate the effect of TIN correction on gene expression. **a** Smoothed scatterplot showing TIN scores and raw read counts for a sample (GSM1722952) with good RNA quality with RIN = 6.7 and medTIN = 71.5 (before correction), (**b**) Smoothed scatterplot showing TIN scores and raw read counts for a sample (GSM1722948) with poor RNA quality with RIN = 2.6 and medTIN = 48.9 (before correction). **c** Smoothed scatterplot showing TIN scores and corrected read counts (using loess regression) for the sample with good RNA quality (after correction). **d**. Scatterplot showing TIN scores and corrected read counts (using loess regression) for the sample with poor RNA quality (after correction). Loess and linear regression trends were indicated as yellow (solid) and red (dashed) curves, respectively

This dependency of gene expression values on TIN scores in low quality RNA samples, if not corrected, can increase the false positive (i.e. Type I error) rates during gene expression analysis. We corrected this bias by normalizing a gene’s raw read count with its corresponding TIN score using a nonparametric locally weighted polynomial regression model (see Methods). As expected, the *loess* correction procedure had little effect on good quality sample (Fig. 6a, c) but effectively neutralized the dependency between read count and TIN score for low quality samples (Fig. 6b, d).

We then explore if we could improve gene expression analysis using TIN corrected gene expression read counts. When comparing 10 high RIN mCRPC samples to 10 low RIN mCRPC samples, we detected 665 differentially expressed genes (DEGs) using the unadjusted gene read count (Additional file 18: Table S5). However, we detected much less DEGs (289) when using TIN-corrected read counts (Additional file 19: Table S6), 172 (60 %) of which were also seen in the unadjusted DEG list (Additional file 20: Figure S14). We performed functional annotation analyses for the 665 DEGs using DAVID [18]. Interestingly, “ribosomal protein” was the most enriched term (adjusted *P* = 3.1 × 10^−16^) (Table 1). We observed the same set of enriched terms when using DEGs detected by comparing GBM samples with RIN = 10 to RIN = 4 (adjusted *P* = 1.7 × 10^−41^) (Table 1; Additional file 21: Table S7). Ribosomal RNAs were expected to be differentially expressed between high RIN samples and low RIN samples, because they were differentially degraded as reflected by the RIN scores. Therefore, most DEGs related to “ribosomal protein” were arguably the false positives due to differential RNA degradation. As a comparison, we also performed functional annotation analysis for the 289 DEGs detected from TIN-adjusted read count. The “*ribosome*” term was completely removed from the enrichment results and replaced with several pathways that were strongly relevant to cancer development and progression such as “*icosanoid metabolic process*”[19], “*fatty acid metabolic process*” [20, 21], and “*prostaglandin metabolic process*”[22] (Table 1). It is noteworthy that these cancer specific pathways were mainly contributed from the 172 common DEGs, while the “ribosome” terms were exclusively contributed from the 493 “unadjusted specific” DEGs. The “TIN-adjusted specific” 117 DEGs were enriched in other pathways that are also highly relevant to cancer, such as “*Purine nucleotide binding proteins*” [23, 24] and “*LIM domain containing proteins*”[25] (Additional file 20: Figure S14).

**Table 1:** Functional annotation analysis using DAVID (http://david.abcc.ncifcrf.gov/) for 4 lists of differentially expressed genes (DEGs)

	Term	P value	Benjamini
Enriched pathways for the 665 differentially expressed genes in mCRPC samples (without TIN correction).	ribosomal protein	7.50E-19	3.10E-16
	ribosome	1.10E-17	4.10E-15
	structural constituent of ribosome	2.00E-17	1.10E-14
	ribosomal subunit	3.70E-16	6.30E-14
	cytosolic ribosome	1.00E-14	1.30E-12
	translational elongation	9.70E-13	1.80E-09
	large ribosomal subunit	5.40E-12	5.10E-10
	ribonucleoprotein complex	1.20E-11	9.40E-10
	translation	7.70E-10	7.10E-07
	cytosolic large ribosomal subunit	7.10E-09	3.80E-07
Enriched pathways for the top 500 differentially expressed genes in human brain Glioblastoma cell line data (without TIN correction).	ribonucleoprotein	9.00E-44	1.70E-41
	structural constituent of ribosome	3.70E-37	1.90E-34
	ribosome	1.80E-34	7.40E-32
	ribonucleoprotein complex	1.20E-32	2.60E-30
	ribosomal subunit	8.80E-30	1.20E-27
	translational elongation	2.80E-28	4.40E-25
	translation	3.70E-26	2.90E-23
	cytosolic ribosome	1.10E-21	9.60E-20
	structural molecule activity	2.70E-20	6.80E-18
	large ribosomal subunit	4.60E-20	2.80E-18
Enriched pathways for the 289 differentially expressed genes in mCRPC samples (after TIN correction).	icosanoid metabolic process	3.10E-05	3.60E-02
	unsaturated fatty acid metabolic process	4.90E-05	2.90E-02
	fatty acid metabolic process	5.60E-05	2.20E-02
	prostaglandin metabolic process	9.20E-05	2.70E-02
	prostanoid metabolic process	9.20E-05	2.70E-02
	Arachidonic acid metabolism	7.80E-04	7.50E-02
	PPAR signaling pathway	2.00E-03	9.60E-02
Enriched pathways for the 117 differentially expressed genes in mCRPC samples (using 3′ tag counting method).	protein homooligomerization	2.20E-03	6.00E-01
	protein complex assembly	8.50E-03	8.30E-01
	protein complex biogenesis	8.50E-03	8.30E-01
	macromolecular complex assembly	1.40E-02	9.10E-01
	protein oligomerization	1.80E-02	9.20E-01
	macromolecular complex subunit organization	2.10E-02	9.20E-01
	cellular macromolecular complex subunit organization	3.40E-01	1.00E + 00

We have shown that TIN correction could significantly reduce false positive DEGS. We next evaluated the performance of TIN correction on false negatives using ERCC spike-in controls from SEQC data as “ground truth”. We removed spike-in transcripts that did not have at least 5 reads in all of the samples. There were 45 transcripts with a set of predetermined fold changes (ranging from 0.67 to 4) between group A and group B. Additional 14 transcripts had identical molar concentration between the two groups. We considered the 45 transcripts as “true positives (TP)” and the 14 transcripts as “true negatives (TN)”. When TIN correction was not applied prior to gene differential expression analysis, 44 out of 45 TPs and 7 out of 14 TNs were called DEGs, resulting in a sensitivity of 0.98 and specificity of 0.5. When TIN correction was applied before gene differential expression, 40 out 45 TPs and 1 out of 14 TNs called as differentially expressed, resulting in a sensitivity of 0.89 and specificity of 0.93 (Table 2). In essence, when using the limited number of spike-in transcripts, TIN correction prior to differential expression analysis decreased its sensitivity from 0.98 to 0.89 but dramatically increased its specificity from 0.5 to 0.93. When measuring the performance by accuracy, TIN correction improved the accuracy from 0.86 to 0.90. In addition, TIN correction moved the estimated fold changes closer to the predetermined fold changes, suggesting that the TIN correction could improve gene quantification (Additional file 22: Figure S15).

**Table 2:** Evaluate TIN correction using SEQC RNA-seq data with spike-in controls

	TIN correction	Without TIN correction
TP	40	44
FN	5	1
Sensitivity	0.89	0.98
TN	13	7
FP	1	7
Specificity	0.93	0.5
Accuracy	0.90	0.86

The qualities of commercially available reference RNA samples used in SEQC project were presumably high. Therefore, the improvement of TIN correction was unlikely to be explained by the mitigation of RNA quality differences. However, in addition to RNA degradation, RNA-seq has many other inherent biases (such as GC content, PolyA selection, mappability, etc) that could also produce non-uniform coverage, which could partially explain the improvement after TIN correction.

### Comparing TIN correction to 3′ tag counting method

**Fig. 7:**
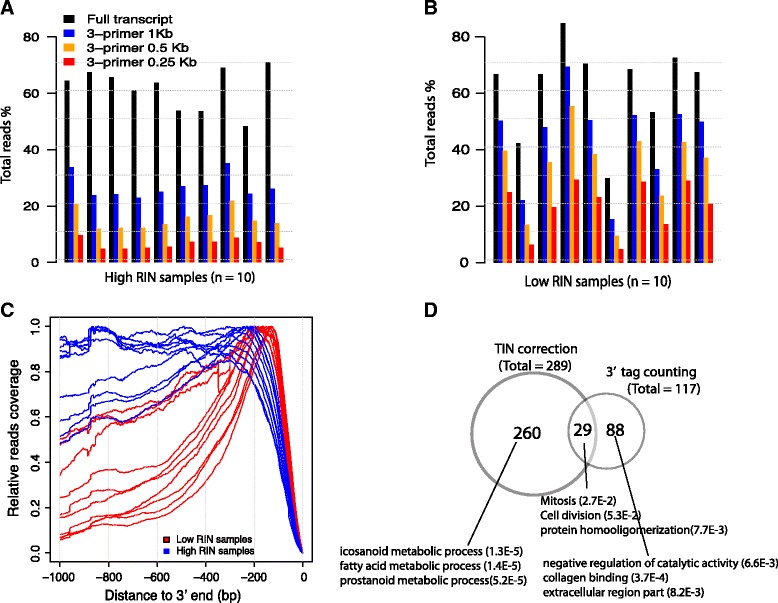
Compare TIN correction to 3′ tag counting method (3TC). **a**-**b** Percentage of retained reads if 3′ 1 Kb, 0.5 Kb and 0.25 Kb were considered. **c** Reads coverage profiles for high RIN (blue) and low RIN mCRPC samples (red). All transcripts were aligned to the 3′ end (i.e transcription end site). **d** Venn diagram showing overlapping between DEGs detected by TIN correction and 3TC

When dealing with RNA-seq data generated from low quality RNA, Sigurgeirsson et al. proposed to use 3′ tag counting (3TC) method to reduce false positives in differential expression analysis [5]. To mitigate the read coverage bias effects on gene expression quantification, 3TC only considered 3′ part of the transcripts by extending *N* (0 ≤ *N* ≤ transcript length) nucleotides from the 3′ end, and all bases and exons beyond *N* length were left out. While 3TC could reduce false positives to some extent, it also reduced statistical power and increased false negatives since only a small fraction of all mapped reads were considered. For example, for 10 mCRPC samples with high RIN values, 61.8 ± 7.5 % of total reads was uniquely mapped to exon regions and can be used for gene expression analysis. However, if 3TC method only considered the 3′ 1 Kb region of transcript, only 26.9 ± 4.2 % of total reads were left to use. And when considered the 3′ 250 nucleotides (see below), only 6.6 ± 1.7 % of reads were left to use, which was equivalent to leave out 90 % usable reads (Fig. 7a). Because of 3′ bias, the fraction of retained reads for samples with low RIN values was significantly higher than those of high quality samples, but only 20.1 ± 9.1 % reads were retained if 3′ 250 nucleotides were used (Fig. 7b).

For 3TC method, deciding the size of *N* is not straightforward: to retain statistical power, *N* should be as large as possible; however, coverage bias cannot be effectively removed if *N* is too large. To determine the proper *N* size, we generated read coverage profiles for 20 mCRPC samples with all expressed transcripts aligned to the 3′ end (i.e. transcription end site) (Fig. 7c). Based on Fig. 7c, we set *N* to 250 and then performed gene expression analysis using the same procedure (see Methods). As we expected, 3TC method detected 117 DEGs (Additional file 23: Table S8), a much smaller number as compared to 289 DEGs that detected with TIN correction and 665 DEGs detected without TIN correction. Although there were 29 common genes detected by both 3TC and TIN correction methods (Fig. 7d). No prostate or prostate cancer relevant pathways were enriched for the 117 DEG list (Table 1).

### Comparing TIN to mRIN

**Fig. 8:**
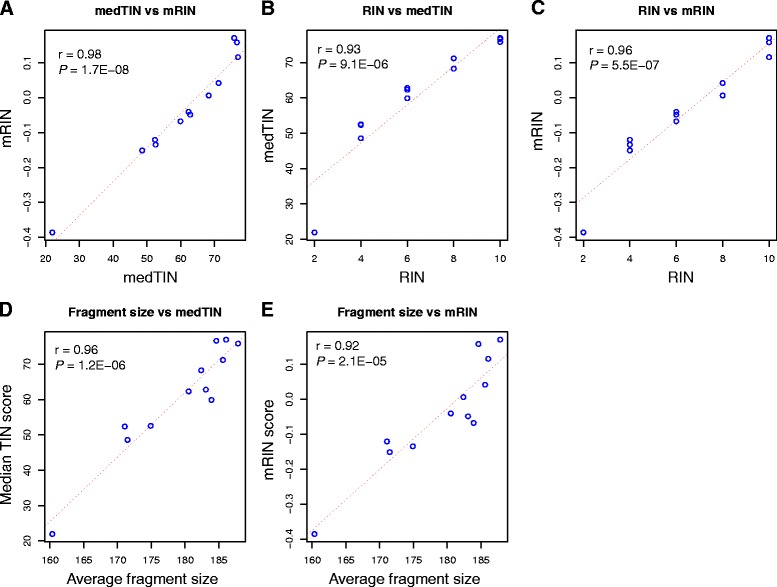
Compare median TIN score (medTIN) with mRIN using 12 GBM RNA-seq data. **a** Concordance between medTIN and mRIN when measuring sample level RNA integrity. **b**-**c** Compare medTIN and mRIN to Agilent’s RIN. **d**-**e** Compare medTIN and mRIN to average RNA fragment size calculated from read pairs. *r* stands for Pearson’s correlation coefficient

When writing this manuscript, we noticed another method named mRIN was also developed to directly assess mRNA integrity from RNA-seq data [26]. Although conceptually similar, mRIN used a modified Kolmogorov-Smirnov (KS) statistic to quantify the 3′ bias of reads coverage while TIN used the Shannon’s entropy. To compare the performance of medTIN and mRIN, we ran mRIN algorithm for the same 12 GBM samples. At sample level, we found medTIN score was highly correlated with mRIN score (*r* = 0.98, *P* = 1.7 × 10^−8^) (Fig. 8a), suggesting the two methods agreed remarkably well despite the underlying computation approaches are different. When comparing mRIN and medTIN to Agilent’s RIN, we found the correlation between mRIN and RIN (*r* = 0.96, *P* = 5.5 × 10^−7^) was slightly better than that of medTIN (*r* = 0.93, *P* = 9.1 × 10^−6^) (Fig. 8b-c). However, when using average RNA fragment size as a benchmark, medTIN (*r* = 0.96, *P* = 1.2 × 10^−6^) performed slightly better than mRIN (*r* = 0.92, *P* = 2.1 × 10^−5^) (Fig. 8d-e). mRIN algorithm also reported GIS (gene integrity score) for each gene. However, we were unable to compare gene level TIN score with GIS, because GIS score was calculated from all samples (in our case, the 12 GBM samples), while TIN was calculated for each gene in each sample. Although GIS is a gene-specific measurement, it is practically less useful than TIN to evaluate gene level integrity since the same gene was often degraded differently in different samples.

## Discussion

Although TIN and Agilent’s RIN are highly concordant, there are three major differences between them. First, RIN is a valuable approach for pre-sequencing sample screening, while TIN scores can only be calculated after RNA-seq data is produced. Second, when using RNA fragment size as a surrogate for RNA integrity to compare RIN and medTIN, we found that Agilent’s RIN only worked well for samples with relative higher RNA integrity, as evidenced by spread of the distribution of blue circles in Fig. 2c. In contrast, medTIN was more sensitive to samples with low integrity, as demonstrated by more spread of distribution of red circles in Fig. 2d. Third, TIN provides RNA quality measurements at transcript level, which not only enables transcript level quality control, but also helps improve gene expression analysis. This is particularly useful given that different genes usually degraded differently.

Since RNA fragment size can be directly estimated from paired-end RNA-seq data, one might question the need for TIN. There are several drawbacks for measuring the RNA integrity using RNA fragment size alone. First, it can only be estimated from paired-end RNA-seq data. Second, RNA fragment size is influenced by other confounding factors such as the fragmentation and size selection steps during library preparation.

We chose 10 mCRPC samples with lower RIN/medTIN scores (low RIN group) and another 10 samples with higher RIN/medTIN scores (high RIN group) with the primary purpose of comparing “RNA degradation effect” on gene expression analysis. Unlike GBM and PBMC datasets that generated from cell lines, the mCRPC dataset was generated from real clinical tissues, and represented the genuine RNA degradation complexity and inter-tumor heterogeneity. However, this was a less than ideal dataset because: 1) these 20 clinical samples were not exact biological replicates and the pathology characteristics of these samples were slightly different (Additional file 14: Table S4). For example, Gleason scores were slightly lower in “low RIN group” (mean = 6.9, median = 7) than that of “high RIN group” (mean = 7.3, median = 8), even though the difference was not statistically significant (*P* = 0.28, two-sided Wilcoxon rank sum test). This pathological differences between low and high RIN group also explained the detection of prostate cancer related DEGs. 2) Unlike SEQC which had spike-in transcripts with predetermined known expression values, there was no “true DEGs” available to accurately test the performance of TIN correction. However, we demonstrated through pathways analysis that TIN correction could remove ribosome genes and identify DEGs that related to prostate cancer.

It is known that oligo(dT) is not a ideal choice for isolating mRNA from degraded samples. Other protocols such as exome capture has been demonstrated with greatly improved performance [27]. However, using oligo(dT) to isolate polyadenylated mRNA is the most widely used RNA-seq protocol especially at the early stage when more advanced protocols are not available. For example, BrainSpan (Atlas of the Developing Human Brain, http://www.brainspan.org/) used oligo(dT) to deplete rRNA during RNA-seq library preparation for RNA samples collected from post-mortem tissues. Being designed to correct non-uniform coverage derived from RNA degradation as well as other biases, our TIN algorithm would be a useful approach to reanalyze or meta-analyze these RNA-seq data available from public repositories. On the other hand, even for samples with reasonable RNA integrity (eg. RIN = 8), 3′ bias still persist (Fig. 1c). And we have demonstrated using the SEQC dataset that TIN could improve gene expression analysis even when the RNA quality is high.

## Conclusions

In this study, we developed TIN as a novel metric to measure RNA integrity, and demonstrated with multiple datasets that the TIN metric is not only a reliable measurement of RNA integrity in both transcriptome and transcript level, but also a valuable metric to neutralize in vitro RNA degradation effect and improve differential gene expression analysis.

## Methods

### RNA-seq datasets

This study used a total of four datasets including three published RNA-seq datasets. All three published datasets were obtained from the NCBI Sequence Read Archive (SRA; http://www.ncbi.nlm.nih.gov/Traces/sra/) or Gene Expression Omnibus (GEO, http://www.ncbi.nlm.nih.gov/geo/). Sequencing reads from all samples were independently aligned to the human reference genome (hg19/GRCh37) using Tophat (v2.0.6) software configured with default options.

1. Human U-251 MG brain glioblastoma cell lines (GBM) [5]. This dataset has 12 pair-end RNA-seq data files available under SRA accession SRP023548. Samples in this dataset have a wide range of RIN values: three samples with RIN value of 10 (SRR873838, SRR873834 and SRR873822), two samples with RIN value of 8 (SRR879615 and SRR879800), three samples with RIN value of 6 (SRR880232, SRR881272 and SRR880070), three samples with RIN value of 4 (SRR881852, SRR881451, and SRR881672) and one sample with RIN value of 2 (SRR881985). Additional file 1: Table S1 presents the details of this dataset.
2. Human peripheral blood mononuclear cells (PBMC) [4]. This dataset has 20 single-end RNA-seq data files available under SRA accession SRP041955. This dataset was developed to estimate the in vitro degradation at 12 h, 24 h, 48 h and 84 h. Additional file 2: Table S2 presents the details of the samples along with their associated RIN values (varied from 2.8 to 9.4).
3. Sequencing quality control consortium data set (SEQC) [28]. The Sequencing Quality Control Consortium analyzed samples containing reference RNA. This dataset was downloaded from NCBI Gene Expression Omnibus (GEO) with accession number GSE49712. This SEQC subset has a total of 10 samples. Group A contains 5 replicates (SRR950078, SRR950080, SRR950082, SRR950084 and SRR950086) of the Stratagene Universal Human Reference RNA (UHRR) and Group B has 5 replicates (SRR950079, SRR950081, SRR950083, SRR950085 and SRR950087) of the Ambion Human Brain Reference RNA (HBRR). ERCC (External RNA Controls Consortium) control mix was spiked in both groups at 2 % by volume. This control mixture contains 92 synthetic polyadenylated oligonucleotides of 250-2000 nucleotides in length, which were meant to resemble human transcripts.
4. Human prostate cancer tissue samples (mCRPC). This study was approved by the Mayo Clinic Institutional Review Board and conducted in accordance with the Declaration of Helsinki. We obtained a total of 120 samples from 46 castration-resistant prostate cancer patients. Out of the collected 120 samples, 62 were blood samples, 18 were metastatic rib lesion biopsies and 40 were metastatic bone tissue biopsies. Tissues were snap frozen with liquid nitrogen and RNA was harvested using Rneasy Plus Mini Kit (Qiagen). RNA libraries were prepared according to the manufacturer’s instructions for the TruSeq RNA Sample Prep Kit v2 (Illumina, San Diego, CA). Briefly, poly-A mRNA was purified from total RNA using oligo dT magnetic beads. The purified mRNA was fragmented at 95 °C for 8 min and eluted from the beads. Double stranded cDNA was made using SuperScript III reverse transcriptase, random primers (Invitrogen, Carlsbad, CA) and DNA polymerase I and RNase H. The cDNA ends were repaired and an “A” base added to the 3′ ends. TruSeq paired end index DNA adaptors (Illumina, San Diego CA) with a single “T” base overhang at the 3′ end were ligated and the resulting constructs were purified using AMPure SPRI beads from Agencourt. The adapter-modified DNA fragments were enriched by 12 cycles of PCR using Illumina TruSeq PCR primers. The concentration and size distribution of the libraries was determined on an Agilent Bioanalyzer DNA 1000 chip and Qubit fluorometry (Invitrogen, Carlsbad, CA). Pair-end RNA sequencing was performed using Illumina HiSeq 2500. Additional file 3: Table S3 presents the details of this dataset.

### Determine the RNA integrity number (RIN)

All mCRPC RNA samples were analysed by Agilent Bioanalyzer 2100 before sequencing. Based on the recorded electropherograms, RIN values were calculated according to the algorithm[7] considering four features: “total RNA ratio” (i.e. the fraction of the area in the region of 18S and 28S compared to the total area under the curve), 28S-region height, 28S area ratio and the 18S:28S ratio. RIN values of GBM, PBMC and SEQC RNA samples were obtained from the original publications.

### Algorithm for computing the transcript integrity number (TIN)

We assumed that a systematic in vitro degradation of a transcript would result in areas with shallow read depths. Hence we designed the TIN metric to capture the uniformity of coverage for a given transcript. Given a transcript of *n* nucleotides long and its read coverage at each nucleotide is (*C*_*i*_; *i* = 1,2,…,*n*). the relative coverage (*P*_*i*_) of each nucleotide is calculated as:

$$ {P}_i=\frac{Coverage\  at\ i-th\  position}{Total\  Coverage}=\frac{C_i}{{\displaystyle \sum }{C}_i} $$

with *P*_*1*_ + *P*_*2*_ + *P*_*3*_ + … + *P*_*n*_ = 1. The coverage evenness of a transcript can be measured by Shannon’s entropy:

$$ H=-{\displaystyle {\sum}_{i=1}^n{P}_i\times log{P}_i} $$

If a particular nucleotide position has no read coverage (i.e. *P*_*i*_ = 0), the entropy *H* = *P*_*i*_ × log *P*_*i*_ = 0. *H* is maximized if the coverage is perfectly uniform (i.e. *P*_*1*_ = *P*_*2*_ = *P*_*3*_ = … = *P*_*n*_ = 1/*n*) across the entire length of the transcript. For computational efficiency, we did not use the entire transcripts to calculate the *H*. Instead, we selected *k* equally spaced positions across the transcript from 5′ end (transcription start site) to 3′ end (transcription end site). *k* is an adjustable parameter in our TIN program. To distinguish different transcripts transcribed from the same gene locus, all the exon-exon joint positions (*j*) were also taken into calculation:

$$ \widehat{H}=-{\displaystyle {\sum}_{i=1}^{\widehat{n}}{P}_i\times log{P}_i = -{\displaystyle {\sum}_{i=1}^{k+j}{P}_i\times log{P}_i}} $$

Although Shannon’s *H* is a useful index to measure the uniformity, its logarithmic scale is difficult to interpret and compare [29]. We addressed this issue by converting the *H* index into real “uniformity” (*U*) as suggested by Jost et al. [29]:

$$ U={e}^{\widehat{H}}=e\left(-{\displaystyle {\sum}_{i=1}^{k+j}{P}_i\times log{P}_{i\ }}\right) $$

where *U* (0 ≤ *U* ≤ (*k* + *j*)) is technically and biologically meaningful since it is equivalent to the number of nucleotides with uniform read coverage. Accordingly, the *TIN* score is the percentage of transcript that has uniform read coverage:

$$ TIN=100\times \frac{U}{\left(k+j\right)}=100\times \frac{e^{\left(-{\displaystyle {\sum}_{i=1}^{k+j}}{P}_i\times log{P}_{i\ }\right)}}{\left(k+j\right)} $$

### Calculating library RNA fragment size

RNA fragment size is the natural measure of the in vitro RNA degradation. Since read pairs were sequenced from both ends of RNA (actually cDNA) fragments, the size of each RNA fragment in the sequencing library can be directly estimated from the distance between read pairs after mapping them to the reference genome. We used uniquely mapped high quality (mapq ≥ 30) read pairs to estimate the RNA fragment size. When a read pair was mapped to the same exon, the fragment size is defined as the genomic distance covered by the two reads (i.e. distance between the “start” of the first read and “end” of the second read). When a read pair was mapped to different exons of the same gene, introns lying between the two reads were subtracted from the genomic distance covered by the read pair. We considered the longest RNA isoform when multiple splicing isoforms (exon skipping, intron retention, alternative donor/acceptor sites, etc.) exist. We removed transcripts with <30 mapped read-pairs to improve the reliability of library fragment size estimation. The “sample level” RNA fragment size was estimated by taking the average of fragment sizes calculated from all read pairs that uniquely mapped to the reference genome. Similarly, the “transcript level” RNA fragment size was estimated from all read pairs that specifically mapped to a transcript.

### Normalizing gene level read counts using TIN metric

For samples with poor RNA quality, both raw read counts and normalized read counts (FPKMs) were positively correlated with TIN scores (see Results). This type of in vitro degradation bias would tamper with gene expression analysis and produce significant numbers of false positives. To correct this bias, we recalibrated the gene level read count using the corresponding TIN score within each sample. In brief, gene level raw read counts *y*_*i*_ (*i* = *1,2,3,…,n. n* is the total number of genes under investigation) were regressed to TIN score *t*_*i*_ using a locally weighted polynomial regression method. For this, we utilized the logarithmic scale of the gene-level counts because it is more robust to outliers that can bias the fit. The R function *loess* was used for the following function.

$$ {y}_i^{\prime }={y}_i-{\widehat{y}}_i+ median\;\left({y}_1,{y}_2,\dots, {y}_n\right) $$

Where *y*_*i*_^′^ denote the normalized read count of gene *i* and *ŷ*_*i*_ denote the fitted value.

### Differential expression analysis

We applied the same procedure for mCRPC dataset (compared 10 samples of lower RIN/TIN values with 10 samples of higher RIN/TIN values), GBM dataset (compared three samples with RIN = 10 to three samples with RIN = 4) and SEQC dataset (compared group A to group B). This method utilized edgeR (version 3.6.8) to perform differential expression analysis [30]. The software was configured to use the TMM (trimmed mean of M values) method for normalizing the library depth differences between samples [31]. Differential expression p-values were FDR corrected using the Benjamini-Hochberg method. Genes with an FDR of ≤ 0.01were considered as differentially expressed between groups.

## Availability of supporting data

Twenty RNA-seq data generated from metastatic prostate cancer tissues were submitted to Gene Expression Omnibus (http://www.ncbi.nlm.nih.gov/geo/) with accession number: GSE70285 (reviewers’ link: http://www.ncbi.nlm.nih.gov/geo/query/acc.cgi?token=knchmaksrfqfnov&acc=GSE70285). Python Code to calculate TIN score (tin.py) is freely available from RSeQC package (www.http://rseqc.sourceforge.net) [32].

## Additional files

Additional file 1: Table S1. Twelve RNA-seq datasets generated from human brain Glioblastoma (GBM) cell line^2^. Accession number, RNA Integrity Numbers (RIN), the median Transcript Integrity Numbers (medTIN), total read pairs, read pairs with mapping quality > 30, and number of genes with at least 10 reads are listed. (XLS 7 kb) Additional file 2: Table S2. Twenty RNA-seq datasets generated from human peripheral blood mononuclear cell (PBMC)^1^. Accession number, RNA Integrity Numbers (RIN), and the median Transcript Integrity Numbers (medTIN), total reads, total reads with mapping quality >30, and number of gene with at least 10 reads are listed. The PBMC samples were stored at room temperature for 0 h, 12 h, 24 h, 48 h and 84 h. Each time point contains 4 individuals (replicates). (XLS 9 kb) Additional file 3: Table S3. 120 RNA-seq datasets generated from clinical tissues of human metastatic castration resistant prostate cancers (mCRPC). Sample ID, RNA Integrity Numbers (RIN), the median Transcript Integrity Numbers (medTIN), total read pairs, read pairs with mapping quality > 30, and number of genes with at least 10 reads are listed. “B” = Blood, “T” = Metastatic soft tumor tissue, “N” = Metastatic bone site, “V1” = Visit 1, “V2” = Visit 2. (XLS 22 kb) Additional file 4: Figure S1. Concordance between RIN and median TIN score for 20 peripheral blood mononuclear cell (PBMC) samples [4]. The PBMC samples were stored at room temperature for 0 h (blue), 12 h (green), 24 h (orange), 48 h (purple) and 84 h (red). Each time point contains 4 individuals (replicates). *r*, Pearson correlation coefficient. (PDF 143 kb) Additional file 5: Figure S2. RIN (RNA integrity number) score distribution for 120 metastatic castration resistant prostate cancer (mCRPC) samples. (PDF 9 kb) Additional file 6: Figure S3. Evaluating RIN and median TIN score using sample level RNA fragment size as benchmark. Only 28 mCRPC samples with RIN < 3 were used. (a) Scatterplot showing relationship between RIN and average RNA fragment size. (b) Scatterplot showing relationship between median TIN score and RNA fragment size. Linear regression lines fitted to data are indicated as black dashed lines. (PDF 186 kb) Additional file 7: Figure S4. Evaluating TIN (x-axis) metric using transcript level RNA fragment size (y-axis) for 12 Glioblastoma (GBM) samples [5]. (a)-(c) Three samples with RIN value of 10 (red); (d)-(e), two samples with RIN value of 8 (purple); (f)-(h) three samples with RIN value of 6 (orange); (i)-(k) three samples with RIN value of 4 (blue); (l) one sample with RIN value of 2 (cyan). Each dot represents 50 transcripts. Black curves indicate locally weighted polynomial regression curves. *r*, Pearson correlation coefficient. (PDF 1490 kb) Additional file 8: Figure S5. Barplot showing Pearson correlation coefficients between TIN and RNA fragment size. 12 Glioblastoma (GBM) samples were stratified by RIN score; RIN = 10 (red), RIN = 8 (purple), RIN = 6 (orange), RIN = 4 (blue) and RIN = 2 (cyan). (PDF 120 kb) Additional file 9: Figure S6. Smoothed scatter plots showing correlation between TIN score and transcript size. (a)-(c) three samples with RIN value of 10; (d)-(e), two samples with RIN value of 8; (f)-(h) three samples with RIN value of 6; (i)-(k) three samples with RIN value of 4; (l) one sample with RIN value of 2. Blue, orange and red represents low, median and high density of data points, respectively. Transcripts with no read coverage or smaller then 100 nucleotide were removed. *r*, Pearson correlation coefficient. Linear regression lines fitted to data are indicated as black dashed lines. (PDF 6875 kb) Additional file 10: Figure S7. Relationship between CDS (coding DNA sequence) size and TIN score for 12 Glioblastoma (GBM) samples. (a)-(c) three samples with RIN value of 10; (d)-(e), two samples with RIN value of 8; (f)-(h) three samples with RIN value of 6; (i)-(k) three samples with RIN value of 4; (l) one sample with RIN value of 2. *r*, Pearson correlation coefficient. Linear regression lines fitted to data are indicated as black dashed lines. (PDF 7104 kb) Additional file 11: Figure S8. Relationship between 3′UTR (untranslated regoin) size and TIN score for 12 Glioblastoma (GBM) samples. (a)-(c) three samples with RIN value of 10; (d)-(e), two samples with RIN value of 8; (f)-(h) three samples with RIN value of 6; (i)-(k) three samples with RIN value of 4; (l) one sample with RIN value of 2. *r*, Pearson correlation coefficient. *r*, Pearson correlation coefficient. Linear regression lines fitted to data are indicated as black dashed lines. (PDF 7201 kb) Additional file 12: Figure S9. Relationship between 5′UTR (untranslated regoin) size and TIN score for 12 Glioblastoma (GBM) samples. (a)-(c) three samples with RIN value of 10; (d)-(e), two samples with RIN value of 8; (f)-(h) three samples with RIN value of 6; (i)-(k) three samples with RIN value of 4; (l) one sample with RIN value of 2. *r*, Pearson correlation coefficient. *r*, Pearson correlation coefficient. Linear regression lines fitted to data are indicated as black dashed lines. (PDF 7140 kb) Additional file 13: Figure S10. Relationship between GC content (GC-ratio) and TIN score for 12 Glioblastome cell line samples. (a)-(c) three samples with RIN value of 10; (d)-(e), two samples with RIN value of 8; (f)-(h) three samples with RIN value of 6; (i)-(k) three samples with RIN value of 4; (l) one sample with RIN value of 2. *r*, Pearson correlation coefficient. *r*, Pearson correlation coefficient. Linear regression lines fitted to data are indicated as black dashed lines. (PDF 6617 kb) Additional file 14: Table S4. List of 10 low RIN/medTIN mCRPC and 10 higher RIN/medTIN mCRPC samples used for differential expression analysis. “N” = Metastatic bone site, “V1” = Visit 1. Whole datasets are available with accession # GSM1722952. (XLS 97 kb) Additional file 15: Figure S11. Dependency between FPKM (y-axis) and TIN scores (x-axis) for 20 mCRPC samples. (a)-(j) 10 high RIN/medTIN mCRPC samples. (k)-(t) 10 low RIN/medTIN mCRPC samples. FPKM, Fragment Per Kilobase exon per Million mapped reads. *r*, Pearson correlation coefficient. (PDF 12400 kb) Additional file 16: Figure S12. Dependency between FPKM (y-axis) and TIN scores (x-axis) for all 6 Glioblastoma (GBM) samples. (a)-(c) 3 GBM samples with RIN value of 10. (d)-(f) 3 GBM samples with RIN value of 4. FPKM, Fragment Per Kilobase exon per Million mapped reads. *r*, Pearson correlation coefficient. Linear regression lines fitted to data are indicated as red dashed lines. (PDF 3998 kb) Additional file 17: Figure S13. Relationship between expression fold change measured by log2 (FPKM) and TIN fold change. (a) mCRPC dataset. (b) GBM dataset. Linear regression lines fitted to data are indicated as red dashed lines. *r*, Pearson correlation coefficient. (PDF 1315 kb) Additional file 18: Table S5. edgeR detected 665 differentially expressed genes (FDR cutoff = 0.01) in mCRPC samples (without TIN correction). FC = Fold Change; CPM = Count Per Million; FDR = False Discovery Rate. (XLS 97 kb) Additional file 19: Table S6. edgeR detected 289 differentially expressed genes (FDR cutoff = 0.01) in mCRPC samples (after TIN correction). FC = Fold Change; CPM = Count Per Million; FDR = False Discovery Rate. (XLS 45 kb) Additional file 20: Figure S14. Venn diagram showing the overlapping between 665 DEGs (before TIN correction) and 289 DEGs (after TIN correction). DEG, differentially expressed gene. (PDF 101 kb) Additional file 21: Table S7. edgeR detected top 1000 differentially expressed genes (FDR cutoff = 0.01) in GBM samples without TIN correction. FC = Fold Change; CPM = Count Per Million; FDR = False Discovery Rate. (XLS 143 kb) Additional file 22: Figure S15. Comparing fold change estimated from RNA-seq data to predetermined fold change (red dashed line). A total of 15 genes with predetermined fold change of 4 were considered. (PDF 95 kb) Additional file 23: Table S8. edgeR detected top 117 differentially expressed genes (FDR cutoff = 0.01) in GBM samples using 3′ count method (3TC). FC = Fold Change; CPM = Count Per Million; FDR = False Discovery Rate. (XLS 22 kb)

## Abbreviations

CDS: coding DNA sequence
DEG: differentially expression gene
ERCC: external RNA controls consortium
FFPE: formalin-fixed, paraffin-embedded
FPKM: fragments per kilobase of transcript per million mapped reads
GBM: glioblastoma
GEO: gene expression omnibus
HBRR: ambion human brain reference rna
mCRPC: metastatic castration resistant prostate cancer
PBMC: peripheral blood mononuclear cell
RIN: RNA integrity number
SEQC: sequencing quality control consortium
SRA: sequencer read archive
TES: transcription end site
TIN: transcript integrity number
TSS: transcript start site
UHRR: stratagene universal human reference rna
UTR: un-translated region

## References

[1] von Ahlfen S, Missel A, Bendrat K, Schlumpberger M (2007). Determinants of RNA quality from FFPE samples. PLoS One.

[2] Masuda N, Ohnishi T, Kawamoto S, Monden M, Okubo K (1999). Analysis of chemical modification of RNA from formalin-fixed samples and optimization of molecular biology applications for such samples. Nucleic Acids Res.

[3] Botling J, Edlund K, Segersten U, Tahmasebpoor S, Engström M, Sundström M (2009). Impact of thawing on RNA integrity and gene expression analysis in fresh frozen tissue. Diagn Mol Pathol.

[4] Gallego Romero I, Pai AA, Tung J, Gilad Y (2014). RNA-seq: impact of RNA degradation on transcript quantification. BMC Biol.

[5] Sigurgeirsson B, Emanuelsson O, Lundeberg J (2014). Sequencing degraded RNA addressed by 3′ tag counting. PLoS One.

[6] Opitz L, Salinas-Riester G, Grade M, Jung K, Jo P, Emons G (2010). Impact of RNA degradation on gene expression profiling. BMC Med Genomics.

[7] Schroeder A, Mueller O, Stocker S, Salowsky R, Leiber M, Gassmann M (2006). The RIN: an RNA integrity number for assigning integrity values to RNA measurements. BMC Mol Biol.

[8] Yang E, van Nimwegen E, Zavolan M, Rajewsky N, Schroeder M, Magnasco M (2003). Decay rates of human mRNAs: correlation with functional characteristics and sequence attributes. Genome Res.

[9] Beelman CA, Parker R (1995). Degradation of mRNA in eukaryotes. Cell.

[10] van Hoof A, Parker R (1999). The exosome: a proteasome for RNA?. Cell.

[11] Houseley J, Tollervey D (2009). The many pathways of RNA degradation. Cell.

[12] Garneau NL, Wilusz J, Wilusz CJ (2007). The highways and byways of mRNA decay. Nat Rev Mol Cell Biol.

[13] Adiconis X, Borges-Rivera D, Satija R, DeLuca DS, Busby MA, Berlin AM (2013). Comparative analysis of RNA sequencing methods for degraded or low-input samples. Nat Methods.

[14] Brisco MJ, Morley AA (2012). Quantification of RNA integrity and its use for measurement of transcript number. Nucleic Acids Res.

[15] Bauer M, Polzin S, Patzelt D (2003). Quantification of RNA degradation by semi-quantitative duplex and competitive RT-PCR: a possible indicator of the age of bloodstains?. Forensic Sci Int.

[16] Gong X, Tao R, Li Z (2006). Quantification of RNA damage by reverse transcription polymerase chain reactions. Anal Biochem.

[17] Duan J, Shi J, Ge X, Dölken L, Moy W, He D (2013). Genome-wide survey of interindividual differences of RNA stability in human lymphoblastoid cell lines. Sci Rep.

[18] Huang DW, Sherman BT, Lempicki RA (2009). Systematic and integrative analysis of large gene lists using DAVID bioinformatics resources. Nat Protoc.

[19] Nie D, Che M, Grignon D, Tang K, Honn KV (2001). Role of eicosanoids in prostate cancer progression. Cancer Metastasis Rev.

[20] Liu Y (2006). Fatty acid oxidation is a dominant bioenergetic pathway in prostate cancer. Prostate Cancer Prostatic Dis.

[21] Baron A, Migita T, Tang D, Loda M (2004). Fatty acid synthase: a metabolic oncogene in prostate cancer?. J Cell Biochem.

[22] Moreno J, Krishnan AV, Swami S, Nonn L, Peehl DM, Feldman D (2005). Regulation of prostaglandin metabolism by calcitriol attenuates growth stimulation in prostate cancer cells. Cancer Res.

[23] Wierenga RK, Hol WG (1983). Predicted nucleotide-binding properties of p21 protein and its cancer-associated variant. Nature.

[24] Fukumoto M, Amanuma T, Kuwahara Y, Shimura T, Suzuki M, Mori S (2014). Guanine nucleotide-binding protein 1 is one of the key molecules contributing to cancer cell radioresistance. Cancer Sci.

[25] Matthews JM, Lester K, Joseph S, Curtis DJ (2013). LIM-domain-only proteins in cancer. Nat Rev Cancer.

[26] Feng H, Zhang X, Zhang C (2015). mRIN for direct assessment of genome-wide and gene-specific mRNA integrity from large-scale RNA-sequencing data. Nat Commun.

[27] Cieslik M, Chugh R, Wu Y-M, Wu M, Brennan C, Lonigro R (2015). The use of exome capture RNA-seq for highly degraded RNA with application to clinical cancer sequencing. Genome Res.

[28] SEQC/MAQC-III Consortium (2014). A comprehensive assessment of RNA-seq accuracy, reproducibility and information content by the Sequencing Quality Control Consortium. Nat Biotechnol.

[29] Jost L (2006). Entropy and diversity. Oikos.

[30] Robinson MD, McCarthy DJ, Smyth GK (2010). edgeR: a Bioconductor package for differential expression analysis of digital gene expression data. Bioinformatics.

[31] Robinson MD, Oshlack A (2010). A scaling normalization method for differential expression analysis of RNA-seq data. Genome Biol.

[32] Wang L, Wang S, Li W (2012). RSeQC: quality control of RNA-seq experiments. Bioinformatics.

