# Supplementary figures and images for: Measure transcript integrity using RNA-seq data
Source: BMC Bioinformatics. 2016 Feb 3;17:58. doi: 10.1186/s12859-016-0922-z (PMC4739097; doi:10.1186/s12859-016-0922-z)

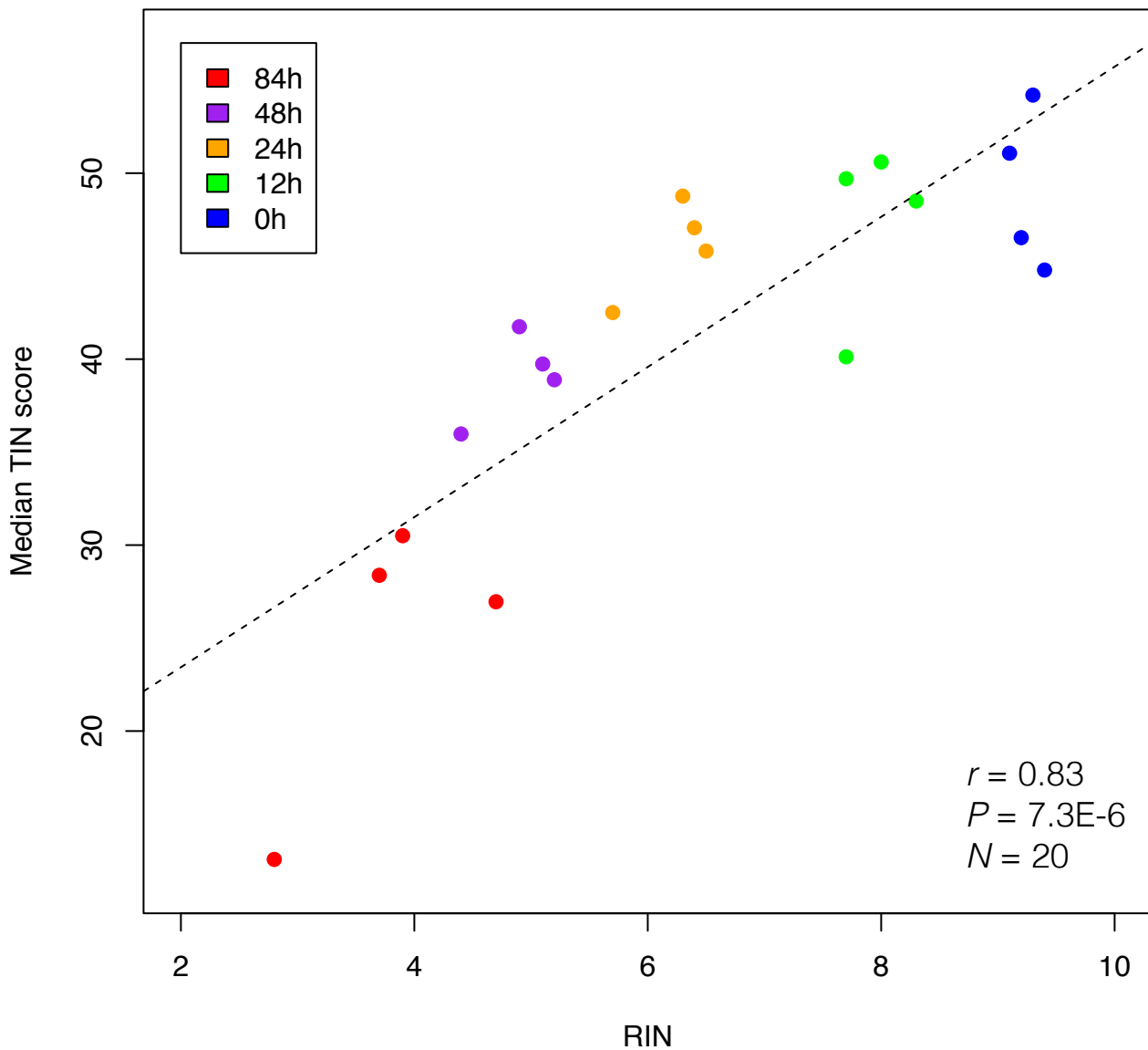

Supplement: Additional file 4: Figure S1. — Concordance between RIN and median TIN score for 20 peripheral blood mononuclear cell (PBMC) samples [4]. The PBMC samples were stored at room temperature for 0 h (blue), 12 h (green), 24 h (orange), 48 h (purple) and 84 h (red). Each time point contains 4 individuals (replicates). r, Pearson correlation coefficient. (PDF 143 kb) [file 12859_2016_922_MOESM4_ESM.pdf]

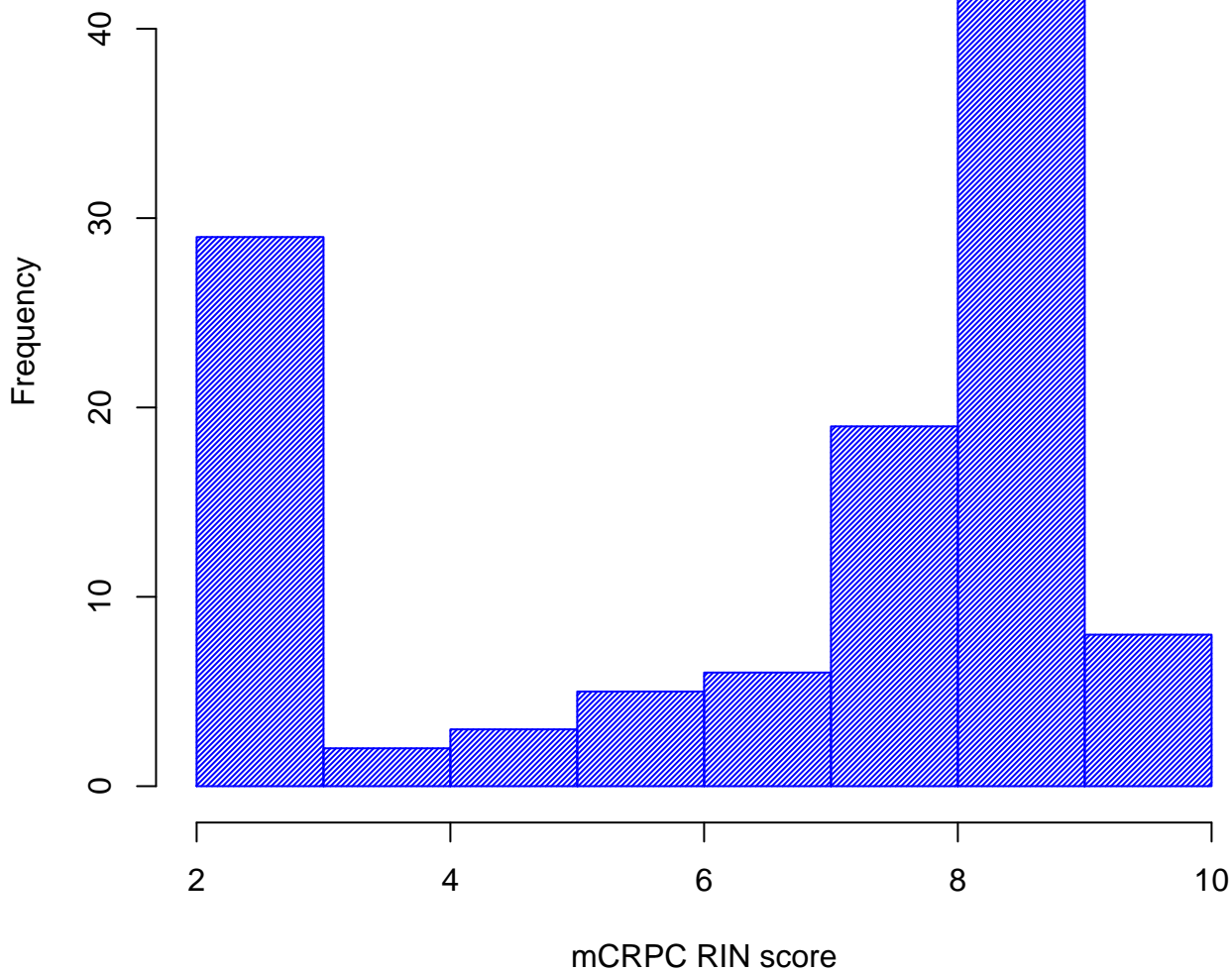

Supplement: Additional file 5: Figure S2. — RIN (RNA integrity number) score distribution for 120 metastatic castration resistant prostate cancer (mCRPC) samples. (PDF 9 kb) [file 12859_2016_922_MOESM5_ESM.pdf]

A

## mCRPC low RIN samples

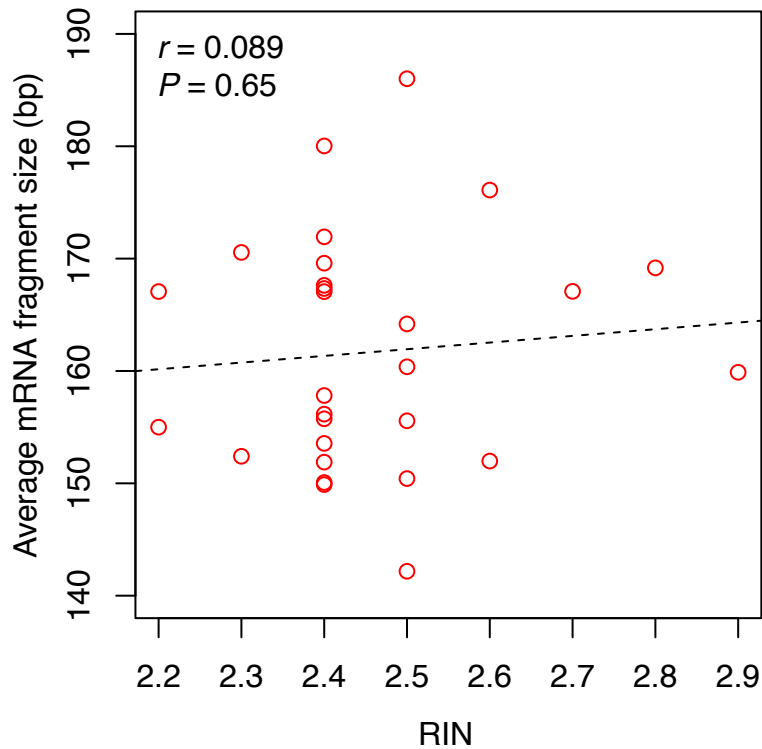

B

## mCRPC low RIN samples

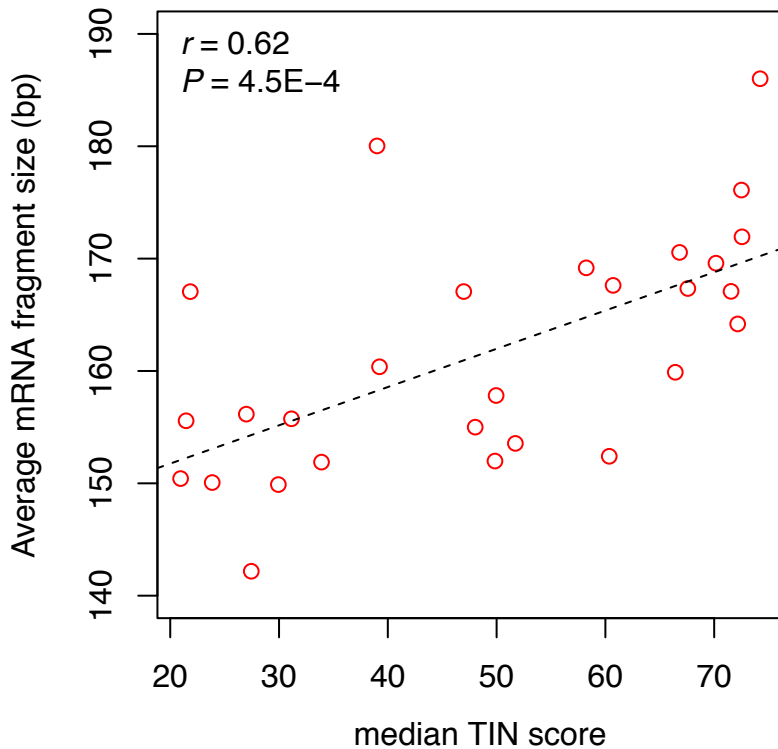

Supplement: Additional file 6: Figure S3. — Evaluating RIN and median TIN score using sample level RNA fragment size as benchmark. Only 28 mCRPC samples with RIN < 3 were used. (a) Scatterplot showing relationship between RIN and average RNA fragment size. (b) Scatterplot showing relationship between median TIN score and RNA fragment size. Linear regression lines fitted to data are indicated as black dashed lines. (PDF 186 kb) [file 12859_2016_922_MOESM6_ESM.pdf]

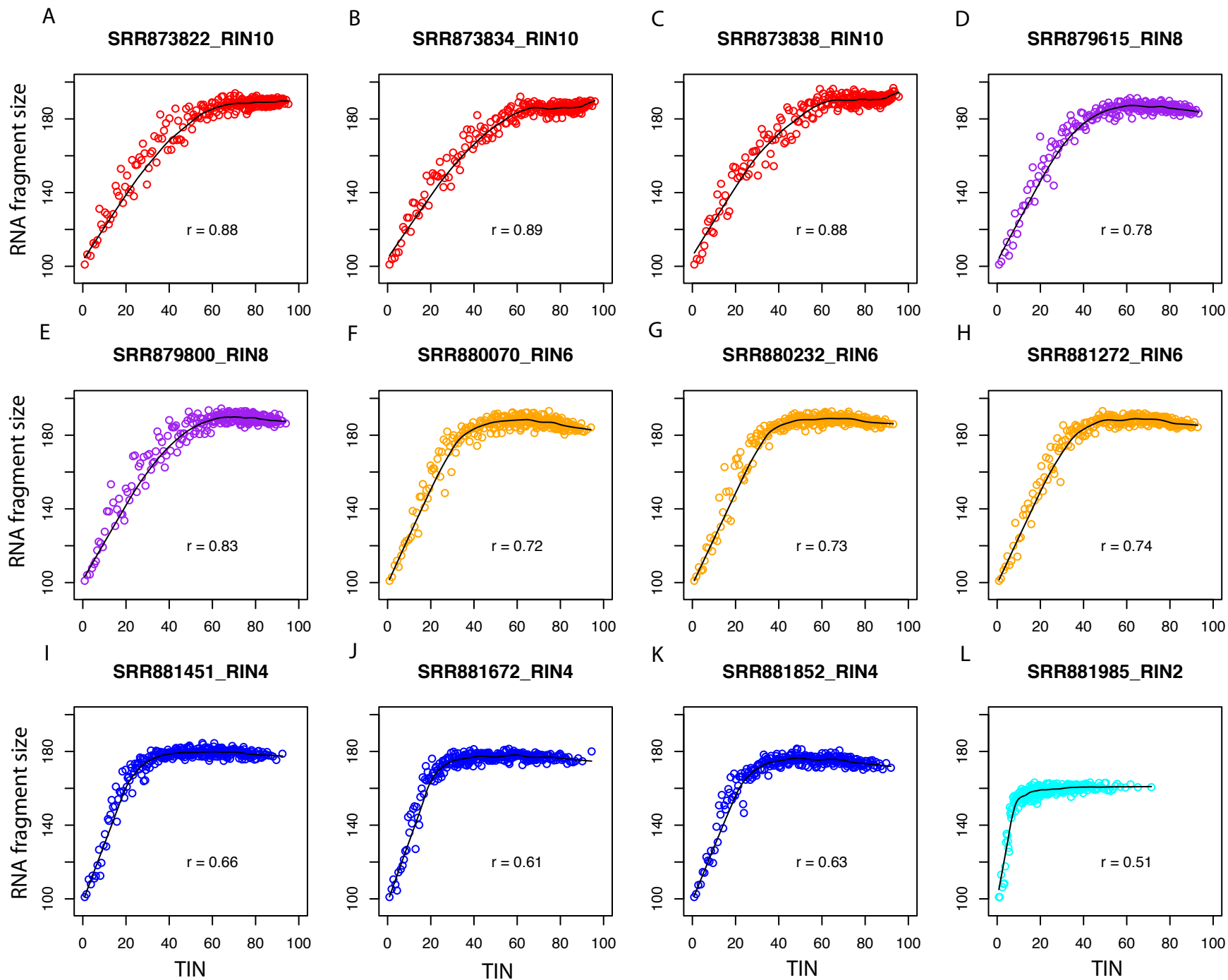

Supplement: Additional file 7: Figure S4. — Evaluating TIN (x-axis) metric using transcript level RNA fragment size (y-axis) for 12 Glioblastoma (GBM) samples [5]. (a)-(c) Three samples with RIN value of 10 (red); (d)-(e), two samples with RIN value of 8 (purple); (f)-(h) three samples with RIN value of 6 (orange); (i)-(k) three samples with RIN value of 4 (blue); (l) one sample with RIN value of 2 (cyan). Each dot represents 50 transcripts. Black curves indicate locally weighted polynomial regression curves. r, Pearson correlation coefficient. (PDF 1490 kb) [file 12859_2016_922_MOESM7_ESM.pdf]

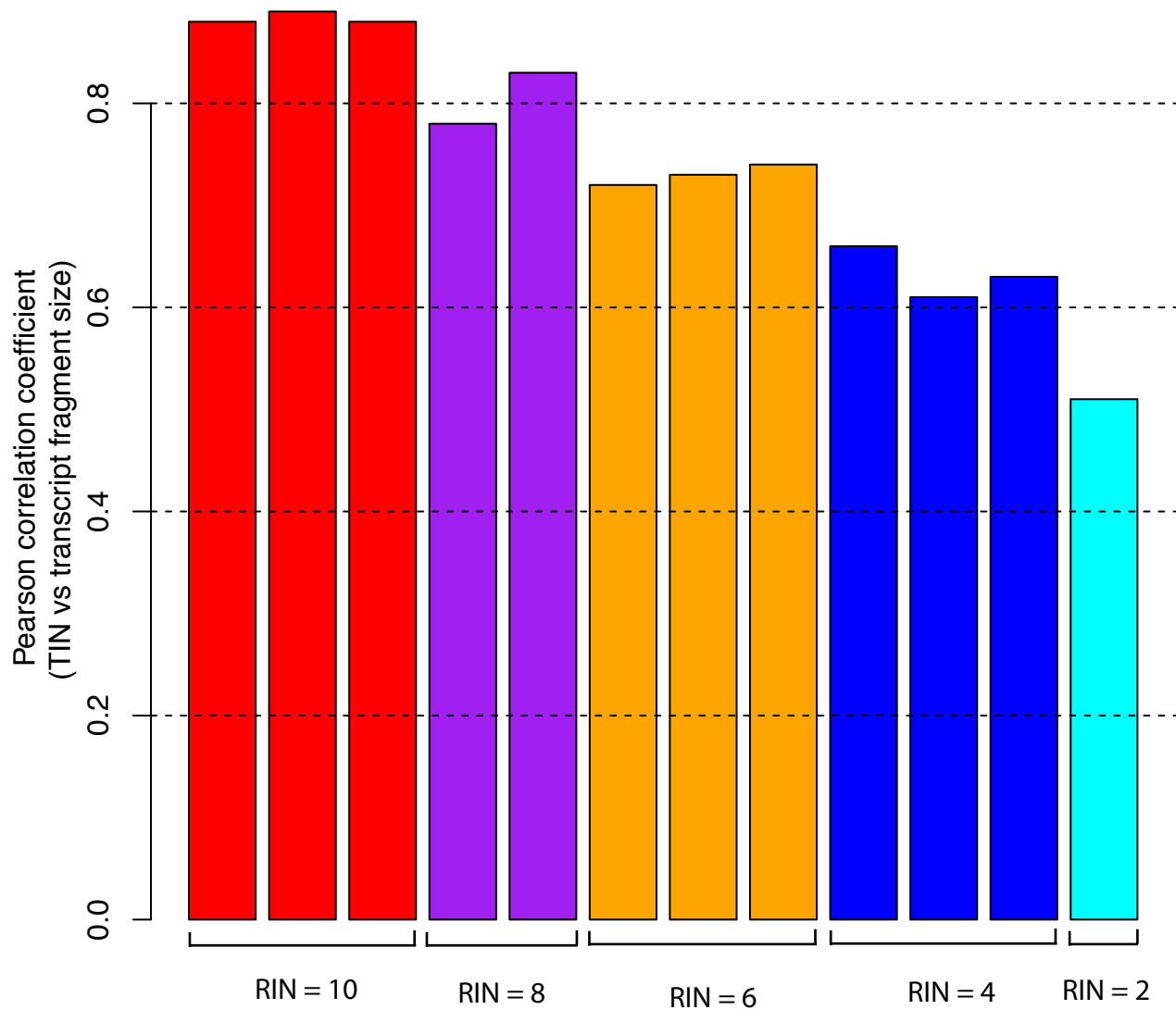

Supplement: Additional file 8: Figure S5. — Barplot showing Pearson correlation coefficients between TIN and RNA fragment size. 12 Glioblastoma (GBM) samples were stratified by RIN score; RIN = 10 (red), RIN = 8 (purple), RIN = 6 (orange), RIN = 4 (blue) and RIN = 2 (cyan). (PDF 120 kb) [file 12859_2016_922_MOESM8_ESM.pdf]

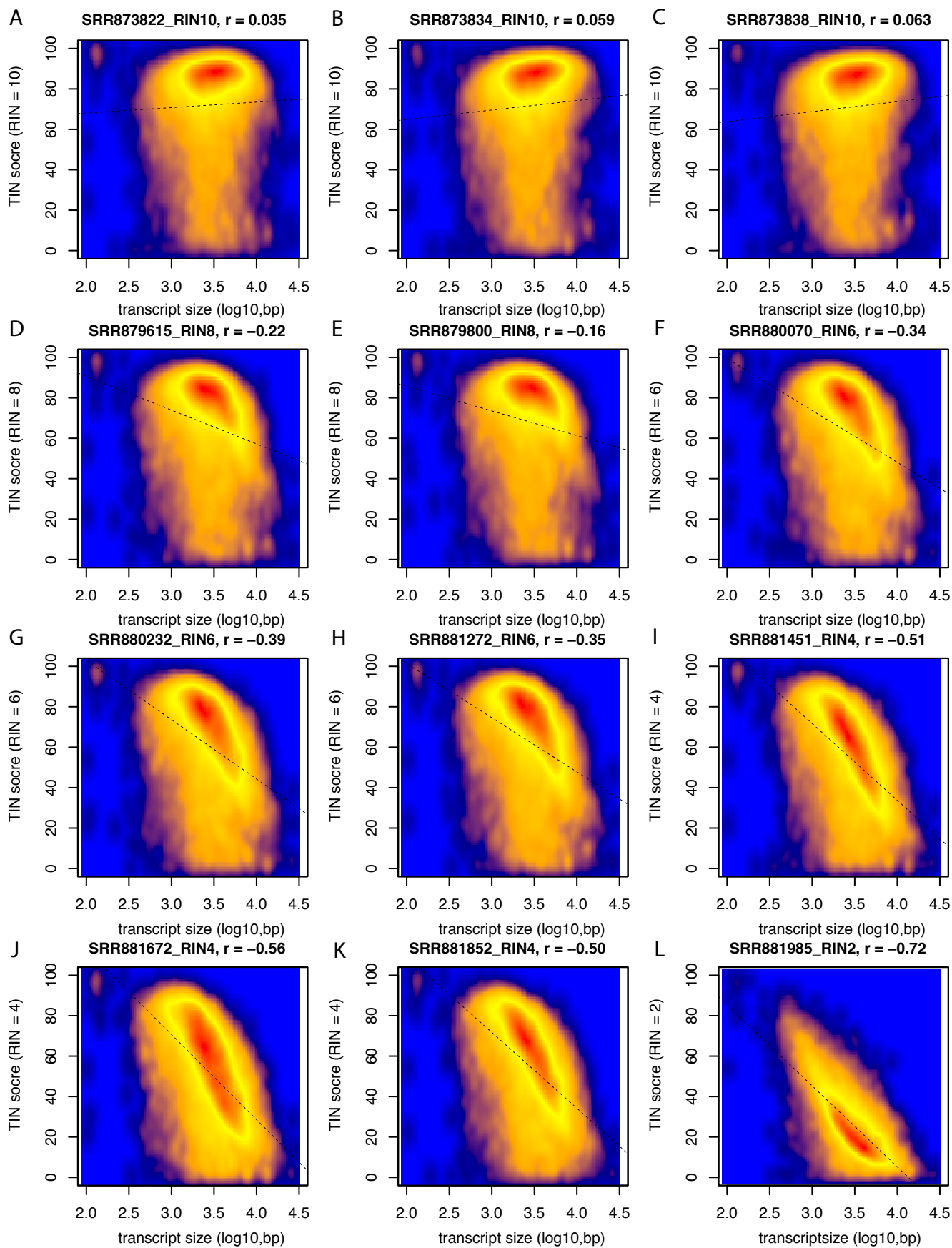

Supplement: Additional file 9: Figure S6. — Smoothed scatter plots showing correlation between TIN score and transcript size. (a)-(c) three samples with RIN value of 10; (d)-(e), two samples with RIN value of 8; (f)-(h) three samples with RIN value of 6; (i)-(k) three samples with RIN value of 4; (l) one sample with RIN value of 2. Blue, orange and red represents low, median and high density of data points, respectively. Transcripts with no read coverage or smaller then 100 nucleotide were removed. r, Pearson correlation coefficient. Linear regression lines fitted to data are indicated as black dashed lines. (PDF 6875 kb) [file 12859_2016_922_MOESM9_ESM.pdf]

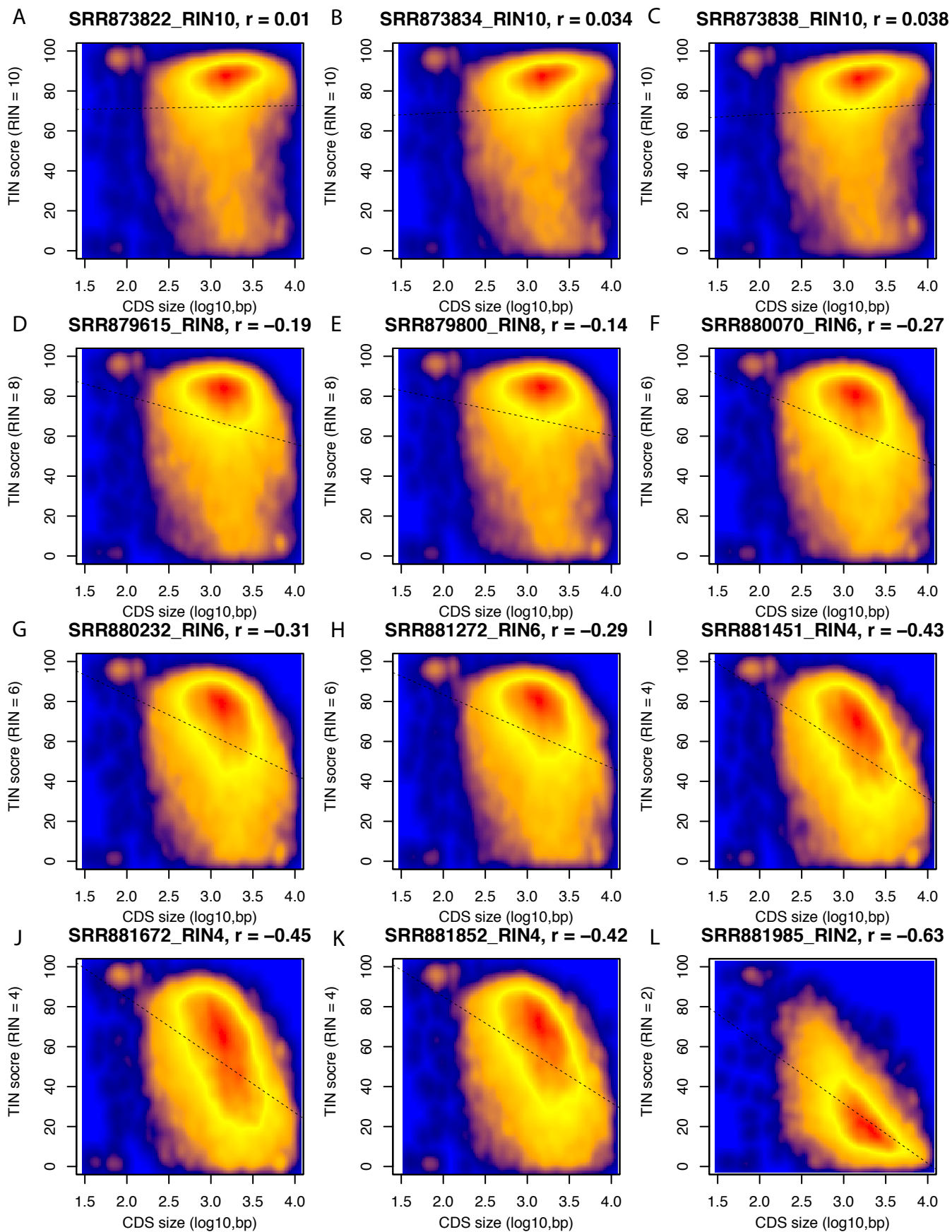

Supplement: Additional file 10: Figure S7. — Relationship between CDS (coding DNA sequence) size and TIN score for 12 Glioblastoma (GBM) samples. (a)-(c) three samples with RIN value of 10; (d)-(e), two samples with RIN value of 8; (f)-(h) three samples with RIN value of 6; (i)-(k) three samples with RIN value of 4; (l) one sample with RIN value of 2. r, Pearson correlation coefficient. Linear regression lines fitted to data are indicated as black dashed lines. (PDF 7104 kb) [file 12859_2016_922_MOESM10_ESM.pdf]

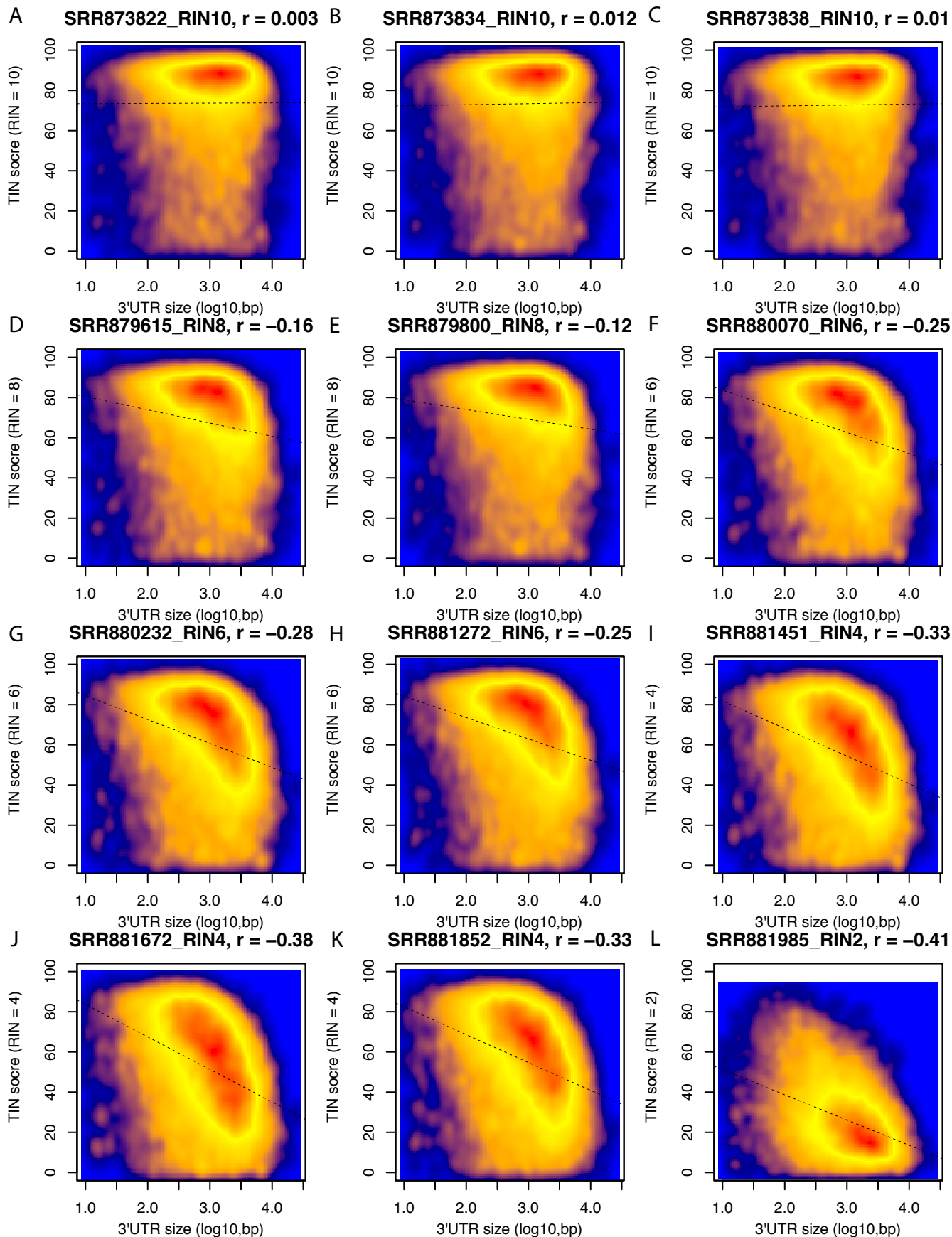

Supplement: Additional file 11: Figure S8. — Relationship between 3′UTR (untranslated regoin) size and TIN score for 12 Glioblastoma (GBM) samples. (a)-(c) three samples with RIN value of 10; (d)-(e), two samples with RIN value of 8; (f)-(h) three samples with RIN value of 6; (i)-(k) three samples with RIN value of 4; (l) one sample with RIN value of 2. r, Pearson correlation coefficient. r, Pearson correlation coefficient. Linear regression lines fitted to data are indicated as black dashed lines. (PDF 7201 kb) [file 12859_2016_922_MOESM11_ESM.pdf]

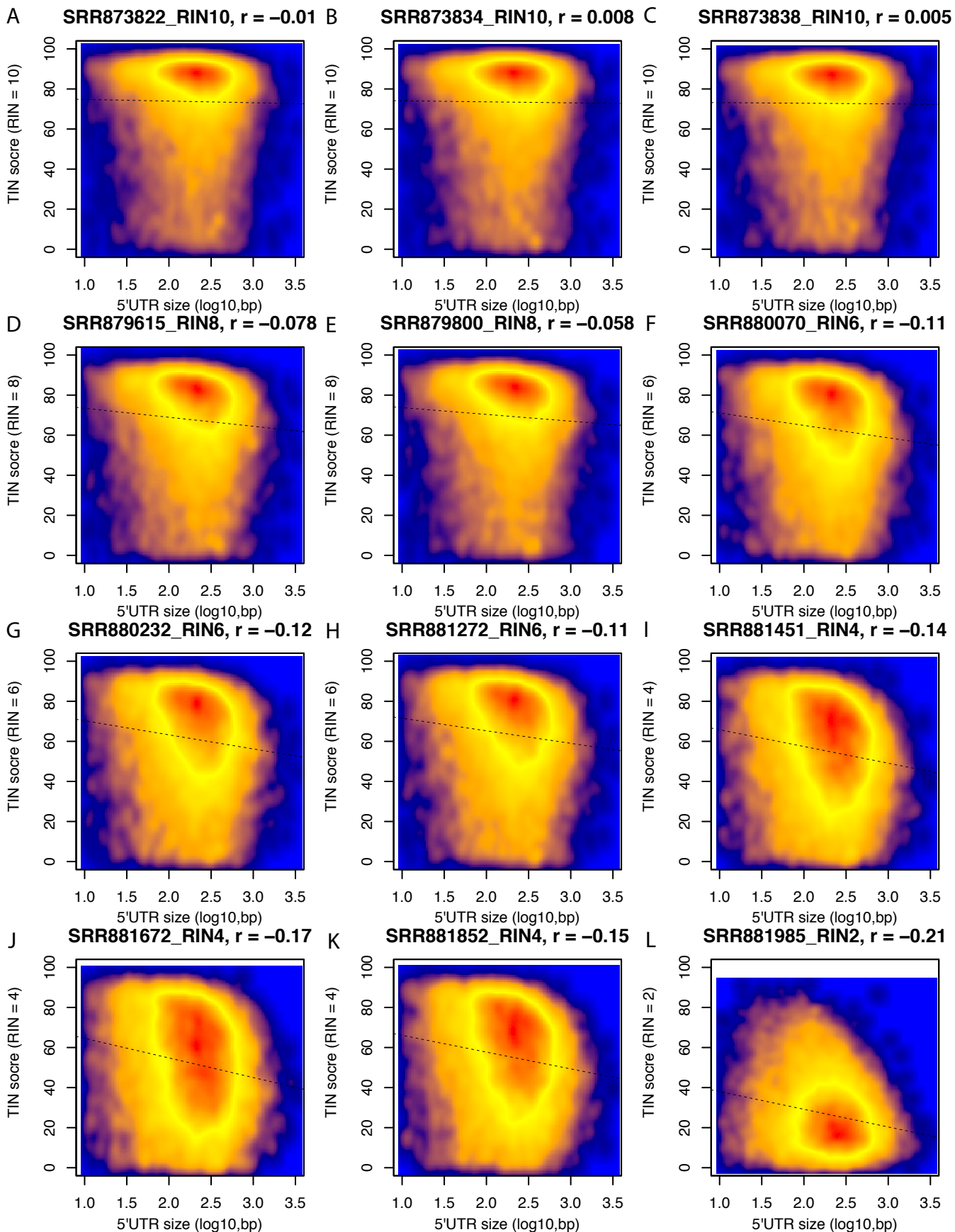

Supplement: Additional file 12: Figure S9. — Relationship between 5′UTR (untranslated regoin) size and TIN score for 12 Glioblastoma (GBM) samples. (a)-(c) three samples with RIN value of 10; (d)-(e), two samples with RIN value of 8; (f)-(h) three samples with RIN value of 6; (i)-(k) three samples with RIN value of 4; (l) one sample with RIN value of 2. r, Pearson correlation coefficient. r, Pearson correlation coefficient. Linear regression lines fitted to data are indicated as black dashed lines. (PDF 7140 kb) [file 12859_2016_922_MOESM12_ESM.pdf]

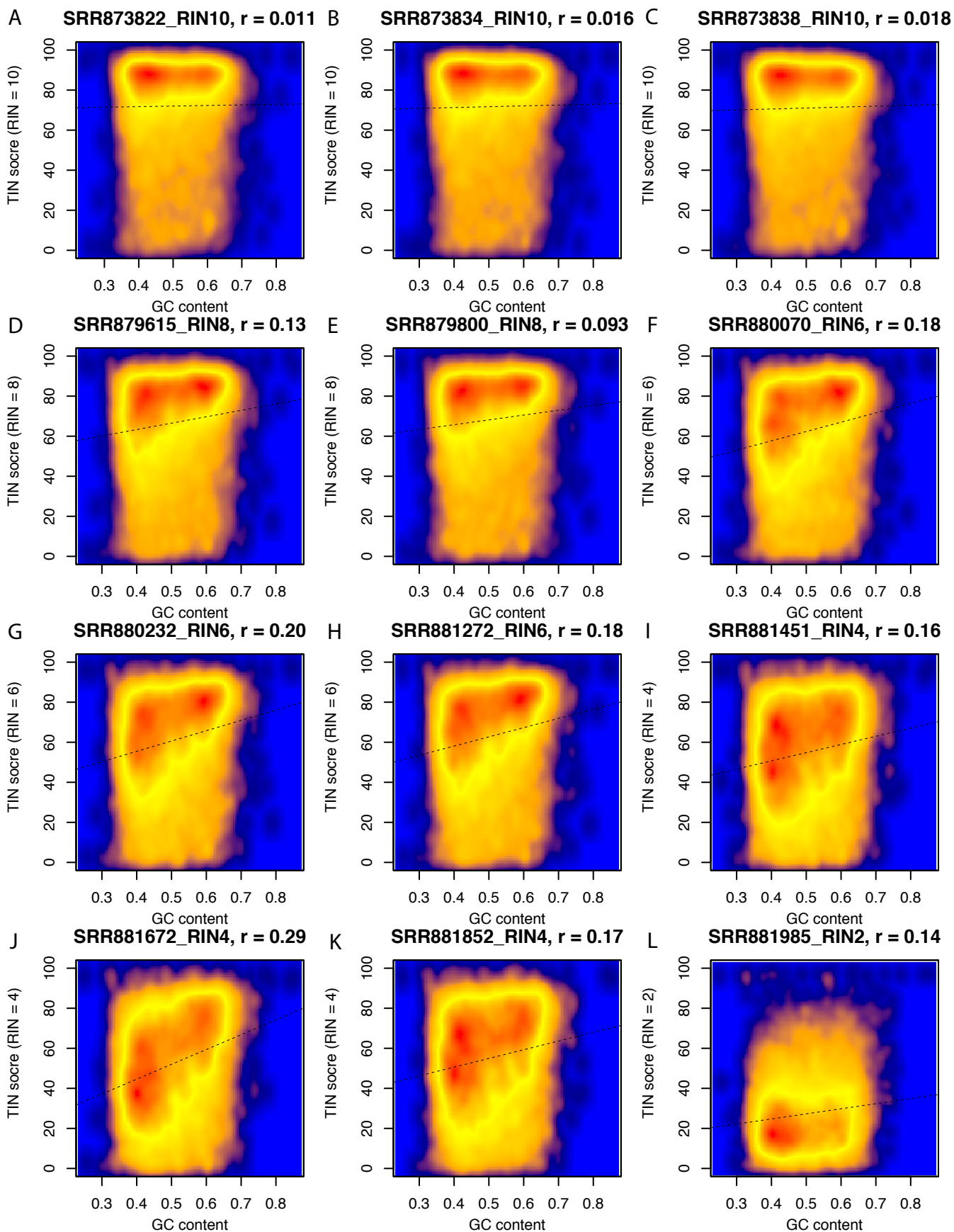

Supplement: Additional file 13: Figure S10. — Relationship between GC content (GC-ratio) and TIN score for 12 Glioblastome cell line samples. (a)-(c) three samples with RIN value of 10; (d)-(e), two samples with RIN value of 8; (f)-(h) three samples with RIN value of 6; (i)-(k) three samples with RIN value of 4; (l) one sample with RIN value of 2. r, Pearson correlation coefficient. r, Pearson correlation coefficient. Linear regression lines fitted to data are indicated as black dashed lines. (PDF 6617 kb) [file 12859_2016_922_MOESM13_ESM.pdf]

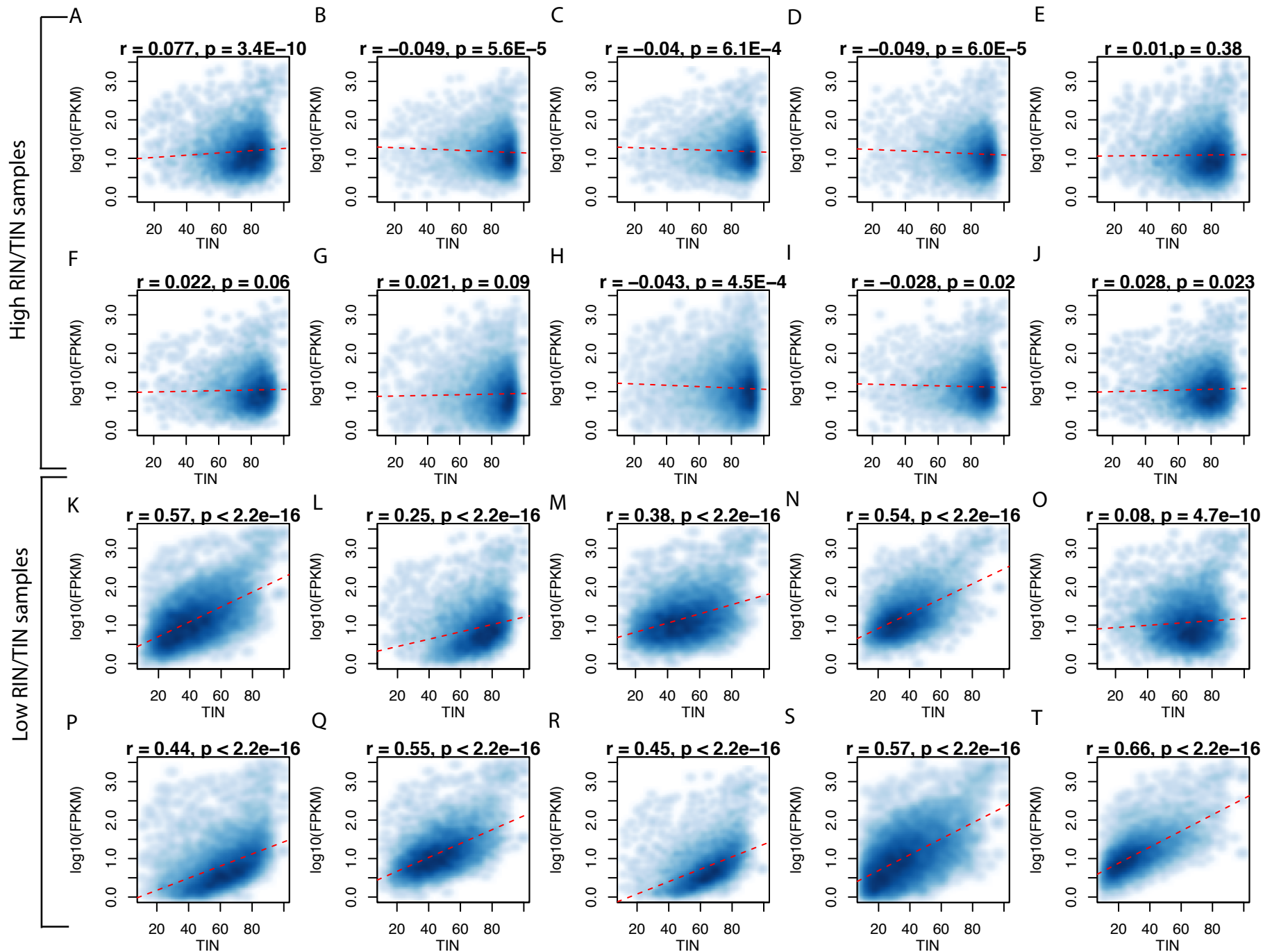

Supplement: Additional file 15: Figure S11. — Dependency between FPKM (y-axis) and TIN scores (x-axis) for 20 mCRPC samples. (a)-(j) 10 high RIN/medTIN mCRPC samples. (k)-(t) 10 low RIN/medTIN mCRPC samples. FPKM, Fragment Per Kilobase exon per Million mapped reads. r, Pearson correlation coefficient. (PDF 12400 kb) [file 12859_2016_922_MOESM15_ESM.pdf]

A

 $r = 0.037, p = 9.4e-5$ 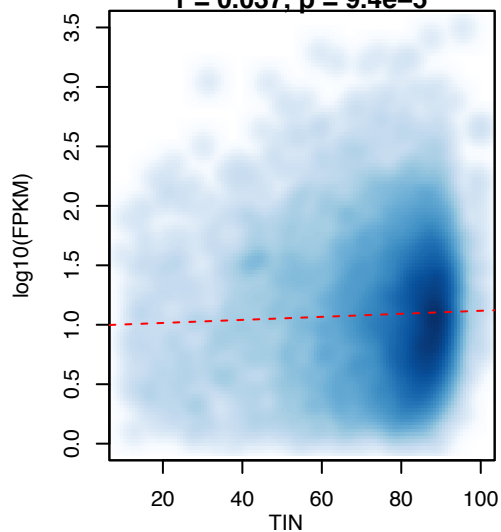

B

 $r = 0.066, p = 5.2e-12$ 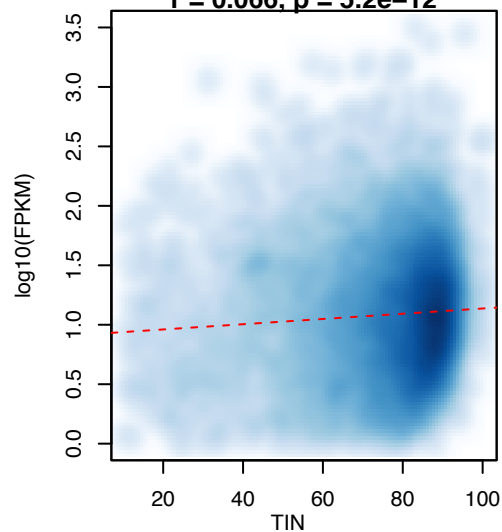

C

 $r = 0.050, p = 1.6e-7$ 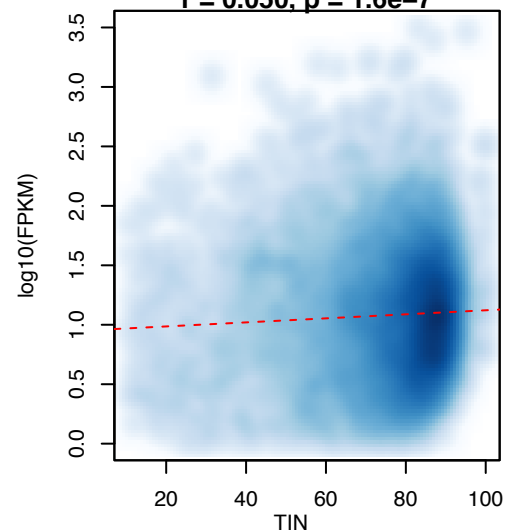

D

 $r = 0.31, p < 2.2e-16$ 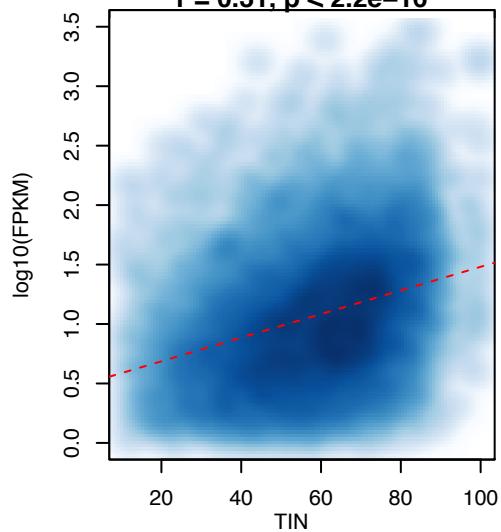

E

 $r = 0.37, p < 2.2e-16$ 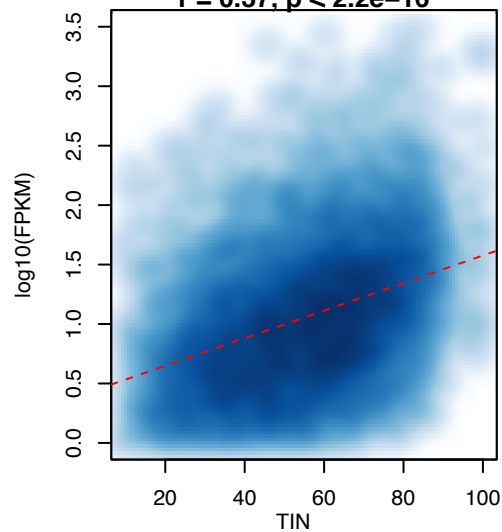

F

 $r = 0.35, p < 2.2e-16$ 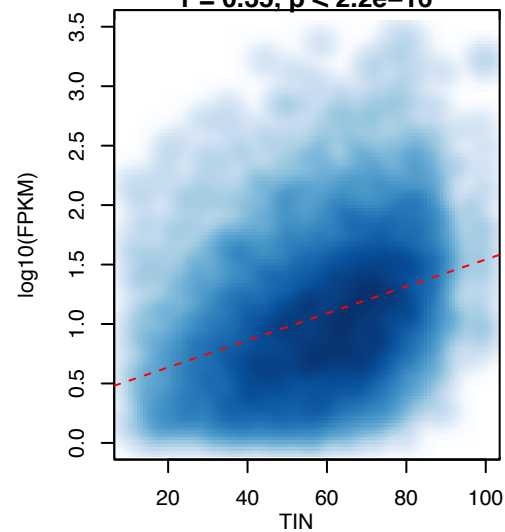

Supplement: Additional file 16: Figure S12. — Dependency between FPKM (y-axis) and TIN scores (x-axis) for all 6 Glioblastoma (GBM) samples. (a)-(c) 3 GBM samples with RIN value of 10. (d)-(f) 3 GBM samples with RIN value of 4. FPKM, Fragment Per Kilobase exon per Million mapped reads. r, Pearson correlation coefficient. Linear regression lines fitted to data are indicated as red dashed lines. (PDF 3998 kb) [file 12859_2016_922_MOESM16_ESM.pdf]

A

mCRPC dataset

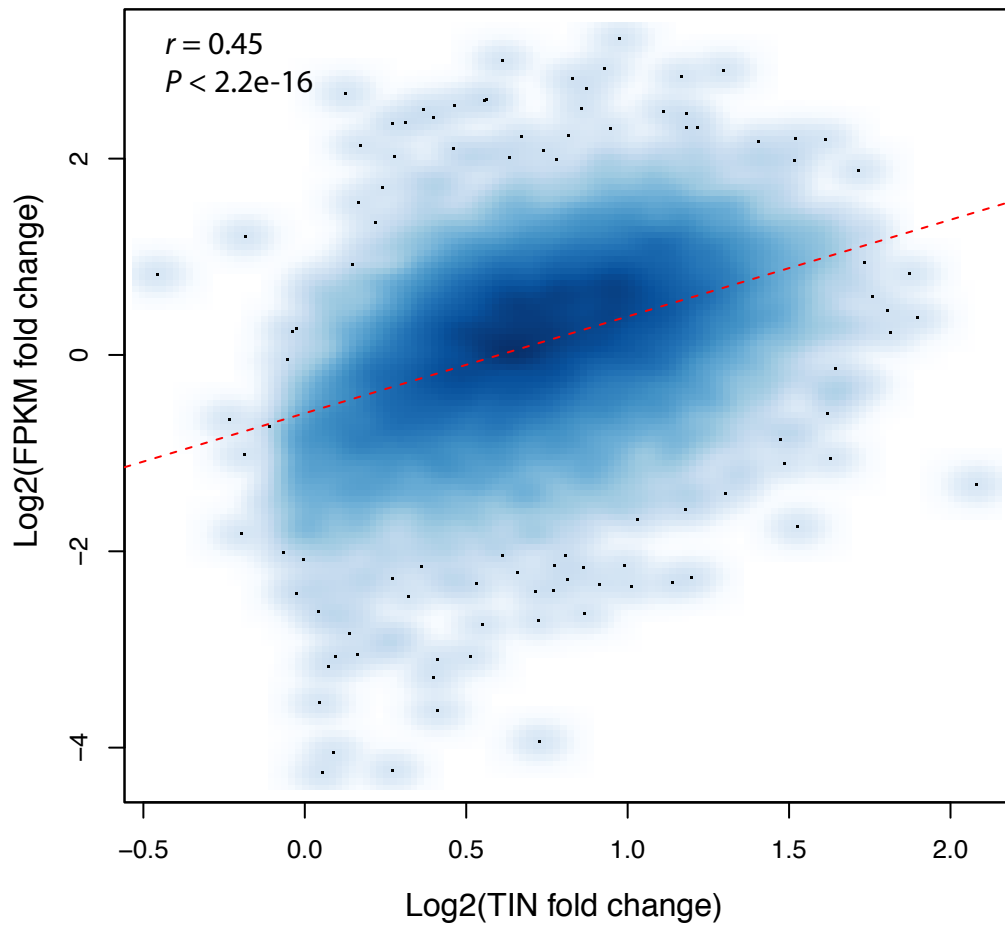

B

GBM dataset

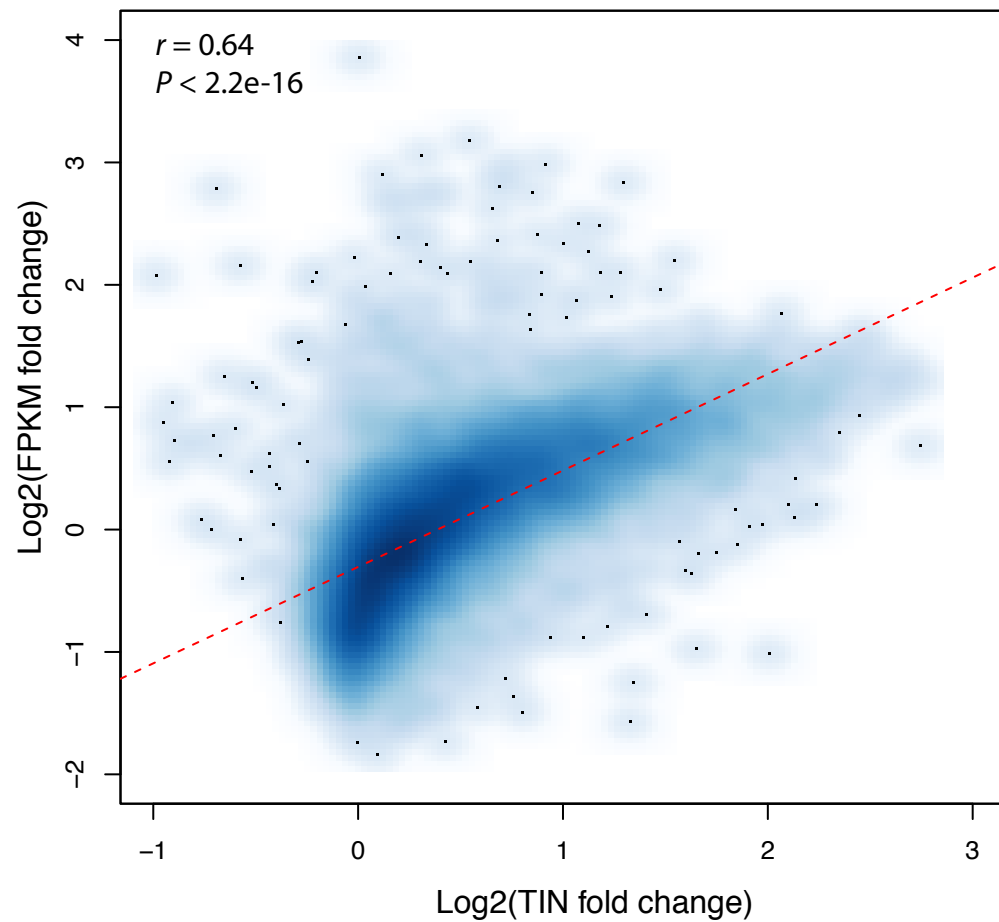

Supplement: Additional file 17: Figure S13. — Relationship between expression fold change measured by log2 (FPKM) and TIN fold change. (a) mCRPC dataset. (b) GBM dataset. Linear regression lines fitted to data are indicated as red dashed lines. r, Pearson correlation coefficient. (PDF 1315 kb) [file 12859_2016_922_MOESM17_ESM.pdf]

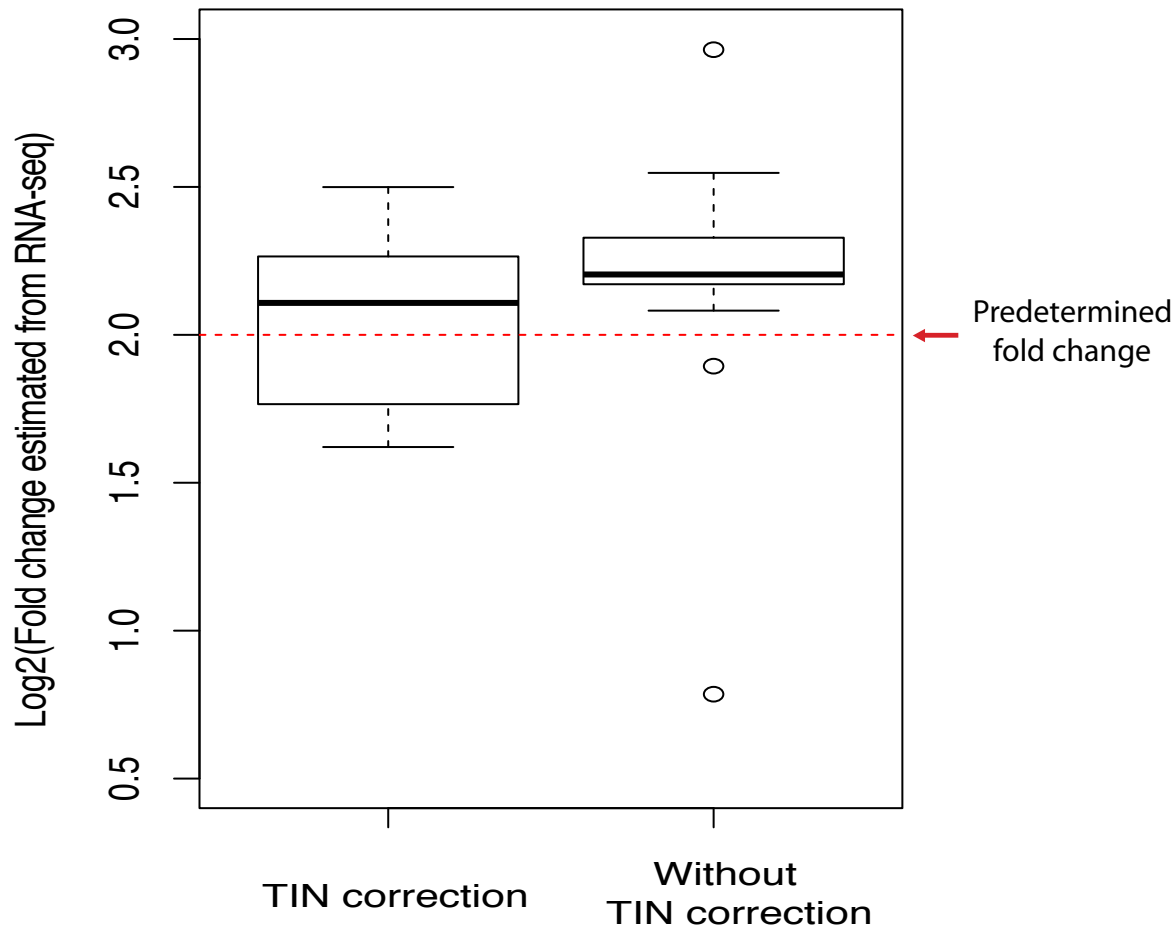

Supplement: Additional file 22: Figure S15. — Comparing fold change estimated from RNA-seq data to predetermined fold change (red dashed line). A total of 15 genes with predetermined fold change of 4 were considered. (PDF 95 kb) [file 12859_2016_922_MOESM22_ESM.pdf]
