# Supplementary material for: Measure transcript integrity using RNA-seq data
Source: BMC Bioinformatics. 2016 Feb 3;17:58. doi: 10.1186/s12859-016-0922-z (PMC4739097; doi:10.1186/s12859-016-0922-z)
Supplement: Additional file 20: Figure S14. — Venn diagram showing the overlapping between 665 DEGs (before TIN correction) and 289 DEGs (after TIN correction). DEG, differentially expressed gene. (PDF 101 kb) [file 12859_2016_922_MOESM20_ESM.pdf]

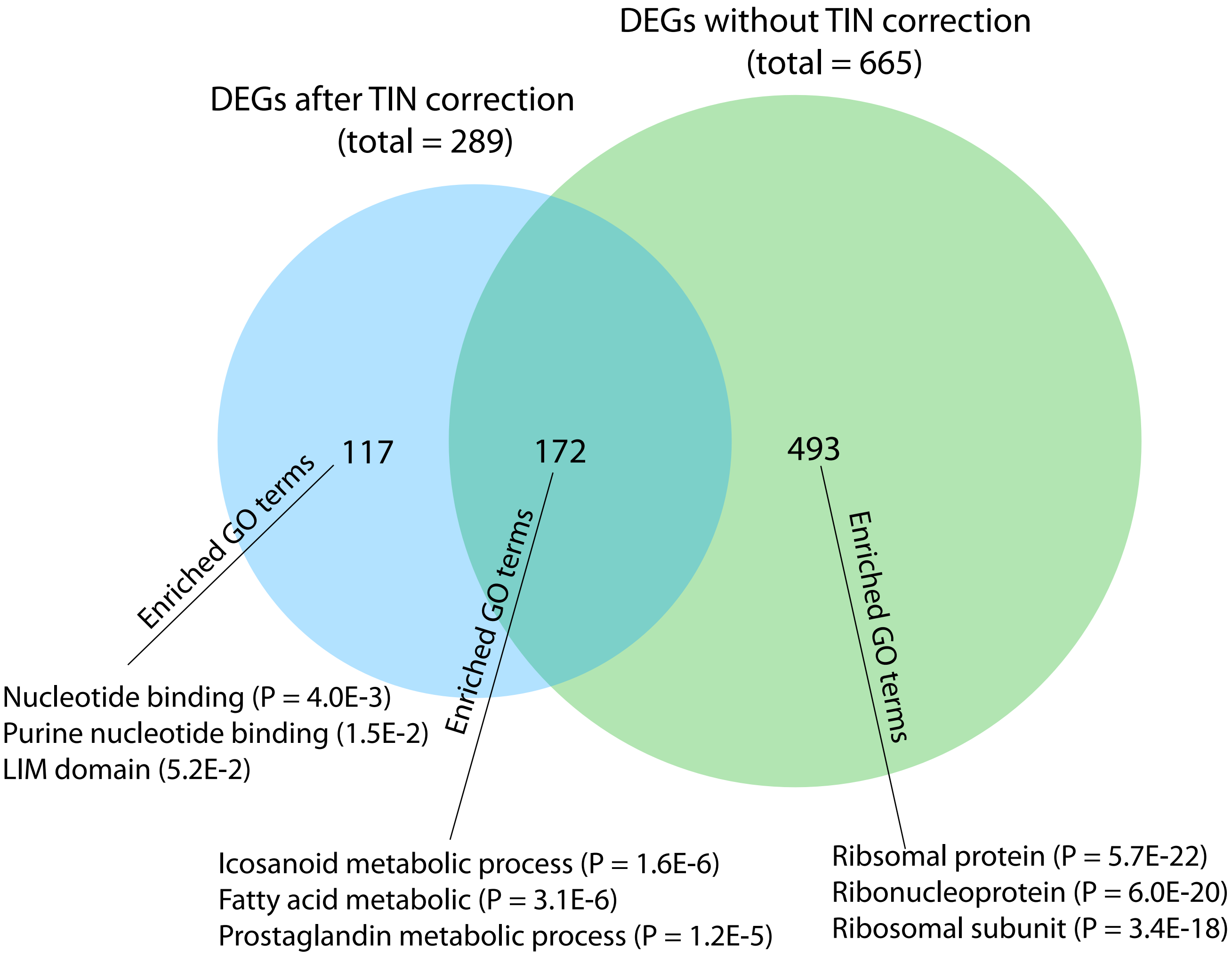
DEGs without TIN correction
(total = 665)
DEGs after TIN correction
(total = 289)
117
172
493
Enriched GO terms
Enriched GO terms
Enriched GO terms
Nucleotide binding (P = 4.0E-3)
Purine nucleotide binding (1.5E-2)
LIM domain (5.2E-2)
Icosanoid metabolic process (P = 1.6E-6)
Fatty acid metabolic (P = 3.1E-6)
Prostaglandin metabolic process (P = 1.2E-5)
Ribsomal protein (P = 5.7E-22)
Ribonucleoprotein (P = 6.0E-20)
Ribosomal subunit (P = 3.4E-18)
